# Stage-specific utility of obesity indices across the chronic kidney disease continuum

**DOI:** 10.1080/0886022X.2026.2646000

**Published:** 2026-03-25

**Authors:** Zhengyang Zhu, Kejun Ren, Xiaowei Duan, Xulei Hu, Yong Lv, Dong Wang, Hua Jin, Lei Zhang, Yiping Wang

**Affiliations:** First Affiliated Hospital, Anhui University of Chinese Medicine, Hefei, Anhui Province, China

**Keywords:** Chronic kidney disease, A Body Shape Index, Triglyceride Glucose–ABSI, NHANES, cardiovascular mortality, mortality prediction

## Abstract

This large-scale study utilizing the US National Health and Nutrition Examination Survey (NHANES, 1999–2018; *n* = 14,175) compared the diagnostic and prognostic utility of two novel obesity indices—A Body Shape Index (ABSI), and Triglyceride Glucose-A Body Shape Index (TyG-ABSI), evaluating their stage-specific utility for CKD risk identification and mortality prediction within the same cohort. TyG-ABSI demonstrated superior performance for identifying prevalent CKD, with the highest quartile (Q4) showing a significantly increased risk after full adjustment (OR = 1.58, 95% CI:1.23–2.03). Conversely, ABSI emerged as a stronger, nonlinear predictor of mortality in established CKD patients. Restricted cubic splines identified significant ABSI z-score thresholds: all-cause mortality risk increased steeply above *z* = 0.624 (HR = 1.44, 95% CI:1.17–1.77), and cardiovascular disease (CVD) mortality above *z* = 0.416 (HR = 1.35, 95% CI:1.09–1.83). After full adjustment, ABSI Q4 remained significantly associated with both all-cause (HR = 1.50) and CVD mortality (HR = 2.40), while TyG-ABSI associations attenuated. ABSI demonstrated superior discriminative accuracy for both all-cause mortality (area under the curve [AUC] = 0.68 vs. TyG-ABSI AUC = 0.66) and CVD mortality (AUC = 0.64 vs. TyG-ABSI AUC = 0.59). Subgroup analyses confirmed the robustness of ABSI for mortality prediction. These findings suggest a shift in the primary driver of adverse outcomes as CKD progresses: TyG-ABSI, capturing metabolic dysregulation, is optimal for identifying CKD risk, whereas ABSI, reflecting anatomical fat distribution, becomes the superior predictor of mortality in established CKD. This differential utility supports stage-specific risk stratification strategies and highlights the broader clinical relevance of obesity phenotyping for cardiovascular risk assessment, metabolic disease screening, and geriatric prognosis.

## Graphical abstract

**Figure:**
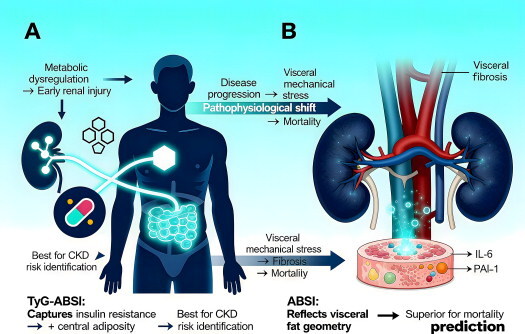

## Introduction

The escalating prevalence of CKD represents a major global health burden, with obesity recognized as a pivotal modifiable risk factor driving both its development and progression [1,2]. Pathophysiological pathways intertwine visceral adiposity, insulin resistance, dyslipidemia, and chronic inflammation, collectively promoting renal injury through glomerular hyperfiltration, endothelial dysfunction, and fibrosis [3,4]. Notably, these pathways are also central to the development of cardiovascular disease, diabetes, and frailty-related outcomes, suggesting that indices capturing these traits may have utility transcending traditional organ-specific boundaries. Obesity-associated metabolic derangements significantly amplify CKD risk beyond traditional factors [5]. Despite the clear modifiable nature of adiposity, accurately identifying individuals at heightened renal risk using clinically feasible metrics remains challenging. Conventional anthropometric indices like BMI and Waist Circumference (WC) provide limited discrimination of metabolically active visceral fat and exhibit poor sensitivity in populations prone to body composition alterations, such as those with advancing CKD [6,7]. While imaging techniques offer precision, their cost, complexity, and limited accessibility restrict routine clinical application [8], underscoring the critical need for practical, low-cost indicators that capture obesity-related metabolic dysfunction linked to renal pathology [9].

Given these limitations, novel indices integrating anthropometry with metabolic parameters have emerged to better characterize obesity phenotypes associated with adverse outcomes [10]. The ABSI, derived from WC, height, and weight, specifically aims to quantify central adiposity and body shape, building upon its original validation by Krakauer et al. where it demonstrated independent prediction of mortality in general populations [11,12]. However, ABSI focuses solely on body geometry and does not directly incorporate metabolic health status. Conversely, the TyG index serves as a robust surrogate marker of insulin resistance, a core mechanism linking obesity to CKD [13,14], as established in foundational studies by Simental-Mendía et al. [15]. The TyG-ABSI, calculated by combining the TyG index with ABSI, represents an integrative approach designed to simultaneously capture central adiposity distribution and underlying metabolic dysregulation [16]. This combined index holds theoretical promise for enhancing risk stratification by addressing both the anatomical and biochemical facets of obesity-related renal injury.

While ABSI has demonstrated utility in predicting cardiovascular outcomes [11,17], its specific performance in CKD risk stratification, particularly against indices incorporating metabolic dysfunction like TyG-ABSI, remains inadequately explored [18]. Existing evidence lacks direct comparison of these indices for predicting both incident CKD and progression in established disease, especially across diverse populations [19]. Determining whether TyG-ABSI offers superior predictive value over ABSI alone is crucial for refining early identification strategies and developing targeted interventions [20].

This study employs the nationally representative NHANES 1999–2018 cohort to conduct a comprehensive comparison between ABSI and TyG-ABSI. First, it contrasts their diagnostic accuracy for identifying prevalent CKD. Second, it evaluates and compares their predictive performance for CKD incidence. Third, it examines their ability to forecast all-cause and CVD mortality within the CKD population [21]. Critically, while prior investigations have often assessed these indices in isolation or focused on singular endpoints, our study is novel in its direct, head-to-head comparison across the CKD continuum—specifically evaluating their differential utility for identifying initial renal risk versus predicting mortality in established disease. The nationally representative sampling strategy ensures external validity across diverse ethnic groups, leveraging the unique advantages of each index—ABSI’s focus on quantifying body shape and central adiposity normalization, and TyG-ABSI’s integration of anthropometric data with metabolic dysregulation (insulin resistance). This analysis aims to establish the comparative clinical utility of these indices, potentially guiding the implementation of more precise risk assessment tools in both primary prevention and CKD management, thereby addressing gaps identified in large CKD epidemiology studies and KDIGO clinical practice guidelines [22–24].

## Methods

### Study design in NHANES

This study utilized a combined cross-sectional and longitudinal cohort design with data from the National Health and Nutrition Examination Survey (NHANES, https://www.cdc.gov/nchs/nhanes) conducted between 1999 and 2018. The assessment of CKD status (prevalent CKD) was performed at a single time point—the NHANES examination visit—constituting a cross-sectional analysis for CKD identification and risk association. The longitudinal component of the design applies exclusively to the analysis of mortality outcomes following the baseline assessment. NHANES, administered by the Centers for Disease Control and Prevention (CDC), is an ongoing serial cross-sectional surveillance program designed to systematically monitor the dietary patterns and health status of the non-institutionalized civilian US population [25]. The survey employs a complex, multi-stage probability sampling design to ensure national representativeness. Data collection is standardized through structured interviews, detailed physical examinations, 24-h dietary recalls, and comprehensive laboratory assessments, including serum biochemistry and urinalysis [26].

**Figure 1.**
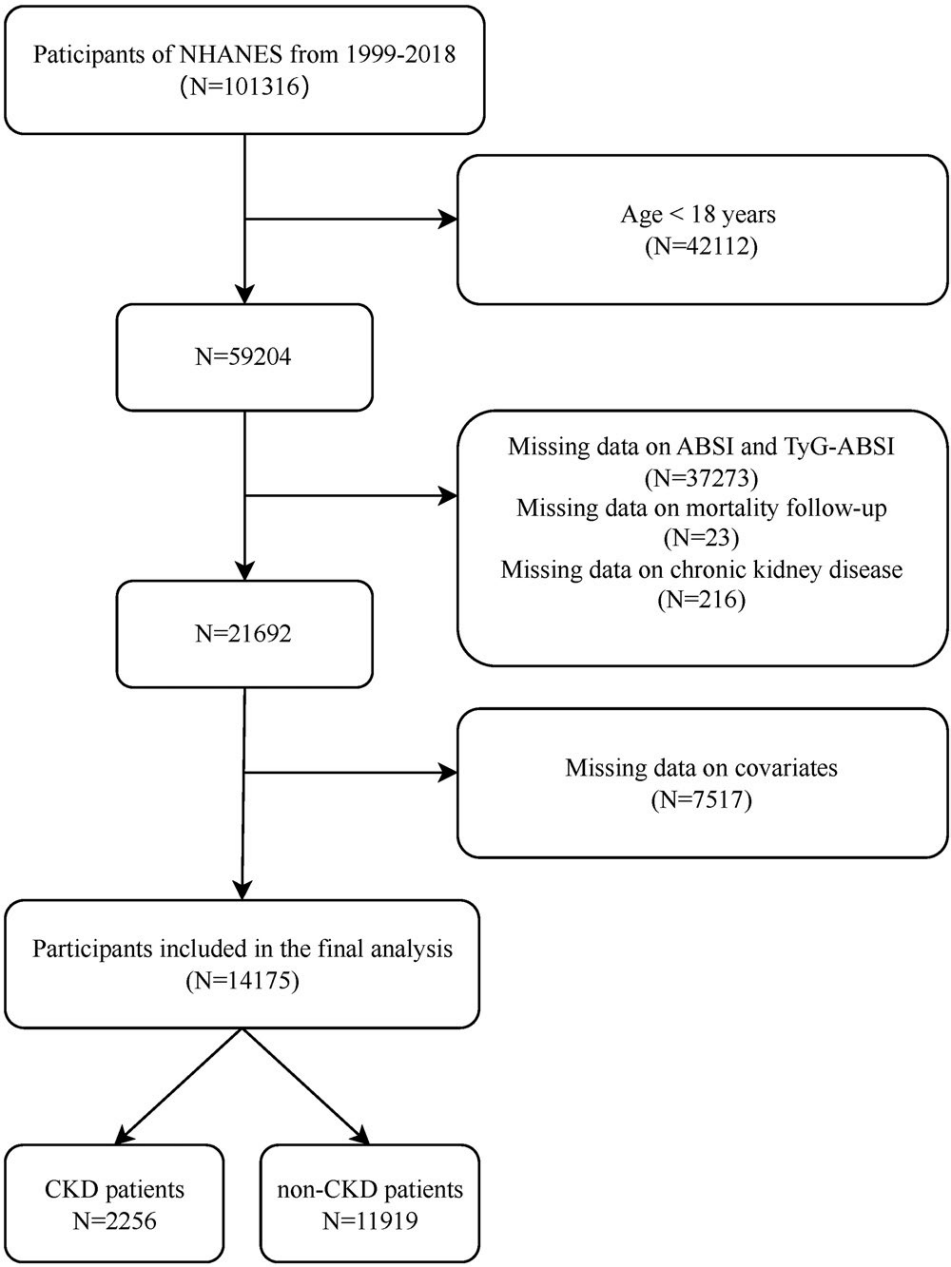
Flowchart of participant selection in NHANES (1999–2018). In the NHANES database, each participant was assigned a unique SEQN identifier. Across different data collection cycles, the same participant’s records were linked and consolidated using their respective SEQN identifiers.

From an initial pool of 101,316 adult participants, we applied a stepwise exclusion process: (1) exclusion of individuals aged <18 years (*n* = 42,112); (2) exclusion of records with missing data for key variables (estimated glomerular filtration rate (eGFR), urine albumin-to-creatinine ratio (UACR), ABSI, TyG-ABSI, or survival outcomes) (*n* = 37,512); and (3) removal of cases with incomplete covariate data (*n* = 7,517). This resulted in a final analytical cohort of 14,175 eligible individuals for the prevalence analysis of CKD. Among this cohort, 2,256 individuals were identified with CKD. This CKD subcohort was subsequently used for the analyses of all-cause mortality and CVD mortality (Figure 1).

### Statement of informed consent

All participants in the original NHANES surveys provided written informed consent. Our study constitutes a secondary analysis of existing, de-identified data from NHANES. Therefore, renewed informed consent was not required for this analysis.

### Assessment of exposure variables in NHANES

Specific anthropometric indices (ABSI, TyG index, and TyG-ABSI) were calculated using the following mathematical formulae. All parameter assessments were performed using standardized methodologies in accordance with established measurement protocols.

$$\text{ABSI}=\frac{\text{WC}}{{\;{\text{Height}}^{1/2}\times \text{BMI}}^{2/3}}$$

$$\text{TyG}=\text{ln}[\frac{\text{TG}\times \text{FBG}}{\;2}]$$

TyG−ABSI=TyG×ABSI

To ensure cross-sex comparability while addressing significant biological sex variations, all continuous anthropometric indices were standardized into sex-stratified z-scores using established normalization procedures. Z-scores transformation was performed by subtracting sex-specific means and dividing by corresponding SD derived from the reference population, ensuring equivalent scaling across male and female subgroups.

$$z-\text{scores}=\frac{x-\mu }{\sigma }$$

Let *x* denote the observed anthropometric measurement value, *μ* represent the arithmetic mean of the corresponding index within sex-stratified reference subgroups, and *σ* indicate the SD derived from gender-specific normative populations. The specific numerical results are in the supplementary file.

### Assessment of outcome variables in NHANES

Prevalent CKD at baseline was defined according to the KDIGO criteria as either: (i) eGFR <60 mL/min/1.73 m^2^ calculated using the CKD Epidemiology Collaboration (CKD-EPI) creatinine equation; (ii) UACR ≥30 mg/g; or (iii) both [23]. eGFR was derived from the CKD-EPI (Equation (1)); Urinary albumin concentration was quantitatively assessed *via* solid-phase fluorescence immunoassay, while urinary creatinine levels were determined enzymatically using a modified Jaffe kinetic method.

$$\text{eGFR}=141*{\text{min}(\frac{\text{Scr}}{\kappa },1)}^{\alpha }*{\text{max}(\frac{\text{Scr}}{\kappa },1)}^{-1.20}*0.993^{\text{Age}}*\text{Sex Factor ∗ Race Factor}$$

**Equation 1:** Scr: Serum creatinine (mg/dL). κ: 0.7 (female), 0.9 (male). α: −0.329 (female), −0.411 (male). Sex factor: 1.018 (female), 1 (male). Race factor: 1.159 (black individuals), 1 (others).

### Covariates in NHANES

This study utilized data collected according to standardized protocols within NHANES. Sociodemographic data were obtained *via* structured household interviews, while medical assessments incorporated standardized physical examinations and laboratory tests. Demographic covariates were defined based on existing research consensus: age, sex (male/female), marital status (married or other [including widowed, divorced, separated, never married, or living with partner]), ethnicity (non-Hispanic White, non-Hispanic Black, Mexican American, or other racial group), educational attainment (less than high school, high school/equivalent, or college or above), and household income stratified by the poverty-to-income ratio (PIR): low income (PIR ≤1.3), middle income (1.3 < PIR ≤3.5), and high income (PIR >3.5). Health-related covariates included hypertension, and hyperlipidemia. Hypertension diagnosis encompassed self-reported diagnosis, antihypertensive medication use, or documented systolic blood pressure ≥140 mmHg and diastolic blood pressure ≥90 mmHg. Hyperlipidemia was defined according to the National Cholesterol Education Program Adult Treatment Panel III (NCEP ATP III) criteria as total cholesterol ≥200 mg/dL, triglycerides ≥150 mg/dL, HDL cholesterol <40 mg/dL for men/<50 mg/dL for women, LDL cholesterol ≥130 mg/dL, or use of lipid-lowering medications. Lifestyle variables included physical activity intensity, categorized as low intensity (light sweating or small increases in breathing/heart rate for at least 10 min over the past 30 days) or high intensity (activity causing heavy sweating or large increases in breathing/heart rate). Smoking status was classified as ‘no’ (<100 cigarettes in lifetime) or ‘yes’ (≥100 cigarettes in lifetime). Alcohol consumption was categorized as ‘no’ (<12 standard drinks per year) or ‘yes’ (≥12 standard drinks per year). Physical examination parameters comprised weight (kilograms), height (centimeters), and BMI, calculated as weight in kilograms divided by height in meters squared. Biochemical parameters included serum creatinine (SCR, µmol/L). All measurements were conducted using standardized laboratory procedures to ensure methodological consistency and data comparability.

### Determination of mortality

All-cause and CVD mortality outcomes were systematically ascertained through probabilistic linkage to the National Death Index (NDI) records [27]. The NDI, a federally maintained mortality database aggregating state death certificate data (available at https://www.cdc.gov/nchs/data-linkage/mortality-public.htm), provided mortality surveillance. Follow-up duration extended from the NHANES examination date until the date of death or December 31, 2019, whichever occurred first. CVD mortality was defined as deaths attributed to cardiovascular or cerebrovascular causes, identified by International Classification of Diseases, Tenth Revision (ICD-10) codes I00-I09, I11, I13, I20-I51, or I60-I69. For brevity, CVD mortality is hereafter termed cardiovascular mortality. Operationally, CVD deaths were identified using values 001 and 005 in the “ucode_leading” field of death records, per the National Center for Health Statistics (NCHS) 2019 Public-Use Linked Mortality File codebook. The final cohort (*N* = 14,175) comprised 2,256 CKD patients and 11,919 non-CKD controls.

### Statistical analysis

All analyses accounted for the complex survey design of NHANES through incorporation of sampling weights, clustering, and stratification, consistent with NHANES analytic guidelines [28]. The reporting of this study follows the STROBE guidelines for observational studies (Supplementary file), and the aspects pertaining to the development and evaluation of the anthropometric indices for risk prediction align with the principles of the TRIPOD statement [29]. Continuous variables were summarized using weighted means with corresponding standard deviations (SD), while categorical variables were reported as frequencies with weighted percentages. Weighted one-way ANOVA (for continuous variables) and Rao-Scott adjusted chi-square tests (for categorical variables) were used to assess differences across baseline characteristic groups, with appropriate application of sampling weights. To address multicollinearity among study variables, variance inflation factors (VIF) were utilized for variable selection; adhering to conventional empirical criteria, VIF > 5 indicates problematic multicollinearity. Given that standard VIF applies to continuous predictors, generalized variance inflation factors (GVIF) were computed to account for the inherent multidimensionality of nominal-scale predictors. To ensure commensurate evaluation of multicollinearity across predictor types and maintain consistency with established VIF thresholds (VIF > 5), VIF was defined as GVIF^(1/(2 × Df)) [30], where Df denotes degrees of freedom (i.e. the number of indicator variables required to represent categorical predictors); this transformation scales the multidimensional GVIF equivalently to standard VIF. All variables—sex, ethnicity, marital status, PIR, smoking status, education level, drinking status, physical activity, hypertension, hyperlipidemia, and age—exhibited VIF values <5 and were retained in the stepwise-adjusted multivariable logistic regression models (Supplementary File, Table S1). To compare associations of ABSI and TyG-ABSI with CKD risk, participants were categorized into quartiles based on their respective z-scores, and weighted multivariable logistic regression models generated stepwise-adjusted OR and 95% CI across four incrementally adjusted models: Model 1 (crude); Model 2 (adjusted for sex and age); Model 3 (further adjusted for race, education level, marital status, PIR, alcohol consumption, smoking, and physical activity); Model 4 (additionally adjusted for health conditions: hypertension and hyperlipidemia). Nonlinear relationships between ABSI and TyG-ABSI with mortality outcomes were explored using RCS with four knots (positioned at the 5th, 35th, 65th, and 95th percentiles of ABSI and TyG-ABSI) [31], generalized additive modeling, and penalized smoothing splines; optimal thresholds for predicting all-cause mortality in CKD patients were identified *via* maximum likelihood estimation. Weighted Cox proportional hazards models were constructed to minimize potential bias from the complex survey design when comparing the independent predictive value of ABSI and TyG-ABSI [32], generating HR with 95% CI across four incrementally adjusted models identical to the logistic regression models. Subgroup analyses and interaction tests examined potential effect modification by covariates, with these stratification factors prespecified as potential effect modifiers; heterogeneity of associations across subgroups was evaluated by incorporating interaction terms into regression models, with statistical significance assessed *via* likelihood ratio tests comparing models with and without these terms. Survival probabilities were estimated using weighted Kaplan-Meier methods, with between-group differences assessed by weighted log-rank tests. To evaluate the association of ABSI and TyG-ABSI with CVD mortality, we employed both standard Cox proportional hazards regression (yielding cause-specific hazard ratios, HR) and Fine-Gray subdistribution hazard models (yielding subdistribution hazard ratios, sHR) to account for non-CVD death as a competing risk. Time-dependent ROC analysis with inverse probability weighting was applied to evaluate predictive performance [33]. Decision Curve Analysis (DCA) was performed to assess clinical net benefit. To provide a more comprehensive assessment of model performance as suggested by the Reviewer, we additionally evaluated model calibration using calibration curves and quantified overall accuracy using the Brier score. The detailed methodologies for DCA, competing risk analysis, calibration curves, and Brier score calculation are provided in the Supplementary File. All analyses were performed using R statistical software (version 4.3.2; R Foundation for Statistical Computing, Vienna, Austria; https://www.R-project.org/) and specialized packages: survey for complex sample analysis, survival for Cox models, cmprsk for competing risk analysis, timeROC for time-dependent AUC calculations, the risk Regression package for calibration and Brier score calculations, and the rmda package for DCA. ggplot2 and survminer for data visualization. Statistical significance was defined as a two-sided p-value < 0.05.

## Results

### Baseline characteristics of NHANES participants

As presented in Table 1, this retrospective cohort study utilizing NHANES data included 14,175 participants (49.94% male, 50.06% female), with a weighted mean age of 46.22 ± 0.29 years. The cohort statistically represents an estimated 79,256,990 US adults, comprising 2,256 CKD patients (representing 9,184,134 US adults) and 11,919 non-CKD controls. Compared to non-CKD individuals, CKD patients exhibited significantly higher SCR (93.87 vs. 74.22, *p* < 0.001), UACR (187.47 vs. 7.36, *p* < 0.001), BMI (29.79 vs. 28.32, *p* < 0.001), ABSI (0.0832 vs. 0.0806, *p* < 0.001), and TyG-ABSI (1.9576 vs. 1.8439, *p* < 0.001), alongside significantly lower height (166.55 vs. 169.99, *p* < 0.001) and eGFR (81.84 vs. 105.26, *p* < 0.001). CKD patients were significantly older (58.45 vs. 44.61 years, *p* < 0.001), while weight showed no significant difference (83.29 vs. 82.06, *p* = 0.106). Demographically, CKD patients had significantly lower male representation (43.44% vs. 50.79%, *p* < 0.001), higher prevalence of less than high school education (21.80% vs. 13.49%, *p* < 0.001), non-married status (40.87% vs. 32.86%, *p* < 0.001), low physical activity (45.58% vs. 31.54%, *p* < 0.001), hypertension (64.70% vs. 31.46%, *p* < 0.001), hyperlipidemia (83.05% vs. 70.86%, *p* < 0.001), and low PIR (25.04% vs. 17.57%, *p* < 0.001). Ethnic distribution differences were significant (*p* < 0.001), with higher representation of Non-Hispanic Black individuals in the CKD group (12.28% vs. 8.69%). All comparisons utilized Student’s t-test for continuous variables and χ^2^-test for categorical variables.

**Table 1.** Weighted baseline participant characteristics by CKD categories.

Variable	Total (*n* = 14175)	Non-CKD (*n* = 11919)	CKD (*n* = 2256)	Statistic	*P*
SCR, Mean (SD)	76.50 (0.28)	74.22 (0.24)	93.87 (1.58)	*t* = 12.37	<0.001
Weight, Mean (SD)	82.21 (0.27)	82.06 (0.27)	83.29 (0.76)	*t* = 1.63	0.106
Height, Mean (SD)	169.59 (0.11)	169.99 (0.12)	166.55 (0.29)	*t* =–10.35	<0.001
BMI, Mean (SD)	28.49 (0.09)	28.32 (0.09)	29.79 (0.24)	*t* = 6.29	<0.001
Age, Mean (SD)	46.22 (0.29)	44.61 (0.28)	58.45 (0.60)	*t* = 24.68	<0.001
UACR, Mean (SD)	28.23 (1.80)	7.36 (0.08)	187.47 (14.84)	*t* = 12.14	<0.001
eGFR, Mean (SD)	102.54 (0.40)	105.26 (0.38)	81.84 (0.98)	*t* =–26.54	<0.001
ABSI, Mean (SD)	0.0809 (0.0001)	0.0806 (0.0001)	0.0832 (0.0001)	*t* = 18.85	<0.001
TyG-ABSI, Mean (SD)	1.8570 (0.0025)	1.8439 (0.0026)	1.9576 (0.0066)	*t* = 17.06	<0.001
Sex, n (%)				χ² = 31.46	<0.001
Male	7056 (49.94)	5980 (50.79)	1076 (43.44)		
Female	7119 (50.06)	5939 (49.21)	1180 (56.56)		
Ethnicity, n (%)				χ² = 25.53	<0.001
Mexican American	2238 (6.98)	1884 (6.91)	354 (7.51)		
Non-Hispanic White	6976 (73.29)	5897 (73.81)	1079 (69.30)		
Non-Hispanic Black	2608 (9.11)	2099 (8.69)	509 (12.28)		
Other	2353 (10.63)	2039 (10.59)	314 (10.91)		
Marital status, n (%)				χ² = 41.73	<0.001
Married	8747 (66.21)	7499 (67.14)	1248 (59.13)		
Other (widowed, divorced, separated, never married, living with a partner)	5428 (33.79)	4420 (32.86)	1008 (40.87)		
PIR, n (%)				χ² = 127.96	<0.001
Low income	4038 (18.43)	3289 (17.57)	749 (25.04)		
Middle income	5325 (35.22)	4365 (34.43)	960 (41.29)		
High income	4812 (46.35)	4265 (48.01)	547 (33.67)		
Smoking, n (%)				χ² = 17.56	0.002
No	7637 (53.38)	6528 (54.02)	1109 (48.53)		
Yes	6538 (46.62)	5391 (45.98)	1147 (51.47)		
Education level, n (%)				χ² = 116.87	<0.001
Less than high school	3217 (14.45)	2519 (13.49)	698 (21.80)		
High school or equivalent	3199 (22.74)	2625 (22.26)	574 (26.45)		
College or above	7759 (62.81)	6775 (64.26)	984 (51.75)		
Drinking, n (%)				χ² = 84.02	<0.001
No	3842 (22.43)	3077 (21.27)	765 (31.30)		
Yes	10333 (77.57)	8842 (78.73)	1491 (68.70)		
Physical activity, n (%)				χ² = 129.26	<0.001
Low physical activity	5219 (33.17)	4094 (31.54)	1125 (45.58)		
High physical activity	8956 (66.83)	7825 (68.46)	1131 (54.42)		
Hypertension, n (%)				χ² = 702.33	<0.001
No	8417 (64.69)	7776 (68.54)	641 (35.30)		
Yes	5758 (35.31)	4143 (31.46)	1615 (64.70)		
Hyperlipidemia, n (%)				χ² = 107.59	<0.001
No	3840 (27.73)	3472 (29.14)	368 (16.95)		
Yes	10335 (72.27)	8447 (70.86)	1888 (83.05)		

All estimates accounted for complex survey designs. NHANES, National Health and Nutrition Examination Survey; CKD, chronic kidney disease; BMI, body mass index; ABSI, A Body Shape Index. TyG-ABSI; Triglyceride Glucose - A Body Shape Index; PIR, poverty-to-income ratio; eGFR, estimated glomerular filtration rate; UACR, urinary albumin-to-creatinine ratio; SCR, serum creatinine; SD, standard deviation; t: t-test; χ^2^: Chi-square test.

### Weighted logistic regression analysis of ABSI and TyG-ABSI with CKD in NHANES

Logistic regression demonstrated significant associations between CKD and standardized ABSI/TyG-ABSI scores (Table 2). For continuous ABSI per 1-SD increase: Model 1 OR = 1.75 (1.64–1.86, *p* < 0.001); Model 4 OR = 1.16 (1.09–1.25, *p* < 0.001). ABSI quartiles in Model 1 showed increasing risk: Q2 OR = 1.27 (1.01–1.61, *p* = 0.042), Q3 OR = 1.94 (1.57–2.40, *p* < 0.001), Q4 OR = 3.64 (3.01–4.39, *p* < 0.001). After full adjustment (Model 4), only Q4 remained significant: OR = 1.37 (1.07–1.76, *p* = 0.014). TyG-ABSI exhibited stronger persistence: Continuous Model 1 OR = 1.74 (1.64–1.85, *p* < 0.001); Model 4 OR = 1.27 (1.16–1.38, *p* < 0.001). Q4 maintained robust significance across all models: Model 1 OR = 3.30 (2.74–3.96, *p* < 0.001); Model 4 OR = 1.58 (1.23–2.03, *p* < 0.001). No significant associations were observed for Q2–Q3 of either index in fully adjusted models.

**Table 2.** Odds ratios for CKD associated with ABSI z-scores and TyG-ABSI z-scores in weighted logistic regression models.

Variables	Model1		Model2		Model3		Model4	
	OR (95%CI)	*P*	OR (95%CI)	*P*	OR (95%CI)	*P*	OR (95%CI)	*P*
**ABSI**								
Continuous	1.75 (1.64–1.86)	<0.001	1.24 (1.16–1.32)	<0.001	1.19 (1.11–1.27)	<0.001	1.16 (1.09–1.25)	<0.001
Quartile								
Q1	1.00 (Reference)		1.00 (Reference)		1.00 (Reference)		1.00 (Reference)	
Q2	1.27 (1.01–1.61)	0.042	1.04 (0.80–1.34)	0.775	1.06 (0.82–1.38)	0.656	1.03 (0.79–1.34)	0.823
Q3	1.94 (1.57–2.40)	<0.001	1.24 (0.98–1.57)	0.071	1.24 (0.97–1.57)	0.083	1.18 (0.93–1.49)	0.167
Q4	3.64 (3.01–4.39)	<0.001	1.57 (1.25–1.99)	<0.001	1.48 (1.16–1.88)	0.002	1.37 (1.07–1.76)	0.014
**TyG-ABSI**								
Continuous	1.74 (1.64–1.85)	<0.001	1.31 (1.21–1.41)	<0.001	1.27 (1.17–1.38)	<0.001	1.27 (1.16–1.38)	<0.001
Quartile								
Q1	1.00 (Reference)		1.00 (Reference)		1.00 (Reference)		1.00 (Reference)	
Q2	1.18 (0.94–1.48)	0.162	0.97 (0.77–1.23)	0.827	1.06 (0.83–1.35)	0.662	1.09 (0.85–1.39)	0.515
Q3	1.59 (1.30–1.94)	<0.001	1.02 (0.83–1.27)	0.835	1.09 (0.87–1.38)	0.451	1.11 (0.88–1.40)	0.392
Q4	3.30 (2.74–3.96)	<0.001	1.59 (1.28–1.97)	<0.001	1.56 (1.23–1.99)	<0.001	1.58 (1.23–2.03)	<0.001

Model 1 (crude adjustment); Model 2 (adjusted for age and sex); Model 3 (further adjusted for ethnicity, education level, marital status, PIR, drinking status, smoking status, and physical activity level); and Model 4 (additionally adjusted for hypertension, and hyperlipidemia). ABSI, A Body Shape Index; TyG-ABSI, Triglyceride Glucose - A Body Shape Index; OR, odds ratio; CI, confidence interval; PIR, poverty-to-income ratio.

### Nonlinear associations of ABSI and TyG-ABSI with mortality

During a median follow-up of 95 months, among 2256 CKD patients, all-cause mortality demonstrated significant associations with renal dysfunction (elevated SCR: 110.08 vs. 86.55, *p* < 0.001; reduced eGFR: 67.29 vs. 88.42, *p* < 0.001), anthropometric alterations (lower weight: 80.48 vs. 84.56 kg, *p* = 0.008; reduced BMI: 28.93 vs. 30.17, *p* = 0.005), advanced age (70.21 vs. 53.13 years, *p* < 0.001), and elevated albuminuria (UACR: 287.02 vs. 142.48, *p* < 0.001). Metabolic derangements including elevated ABSI (0.0853 vs. 0.0823, *p* < 0.001) and TyG-ABSI (2.0501 vs. 1.9157, *p* < 0.001) were observed, alongside demographic disparities including male predominance (50.02% vs. 40.46%, *p* = 0.003), higher Non-Hispanic White representation (78.86% vs. 64.98%, *p* < 0.001), unmarried status (47.82% vs. 37.74%, *p* = 0.003), and lower educational attainment (college+: 43.87% vs. 55.31%, *p* < 0.001). Modifiable risk factors included smoking prevalence (58.24% vs. 48.41%, *p* < 0.001), low physical activity (53.34% vs. 42.08%, *p* < 0.001), and comorbidities (hypertension: 82.98% vs. 56.44%, *p* < 0.001; hyperlipidemia: 87.37% vs. 81.09%, *p* = 0.003). CVD mortality similarly correlated with renal impairment (SCR: 109.04 vs. 92.20, *p* < 0.001; eGFR: 64.70 vs. 83.73, *p* < 0.001), age (72.69 vs. 56.87 years, *p* < 0.001), elevated ABSI (0.09 vs. 0.08, *p* < 0.001) and TyG-ABSI (2.04 vs. 1.95, *p* < 0.001), male sex (55.71% vs. 42.08%, *p* < 0.001), Non-Hispanic White ethnicity (78.30% vs. 68.30%, *p* = 0.005), income disparity (middle income: 52.71% vs. 40.03%, *p* = 0.007), low physical activity (53.41% vs. 44.72%, *p* = 0.031), and hypertension (84.65% vs. 62.49%, *p* < 0.001) (Tables S2–S3).

**Table 3.** The association between sex-specific ABSI and TyG-ABSI z-scores with both all-cause mortality and CVD mortality was analyzed using two-piecewise regression models.

Outcome^a^	HR (95%CI)	*P*
**ABSI**		
All-cause mortality		
Model 1 Fitting model by standard linear regression	1.80 (1.68–1.94)	<0.001
Model 2 Fitting model by two-piecewise linear regression		
Inflection point	0.624	
<0.624	2.09 (1.80–2.42)	<0.001
≥0.624	1.44 (1.17–1.77)	<0.001
P for likelihood test		0.016
**CVD mortality**		
Model 1 Fitting model by standard linear regression	1.83 (1.62–2.07)	<0.001
Model 2 Fitting model by two-piecewise linear regression		
Inflection point	0.416	
<0.416	2.52 (1.83–3.46)	<0.001
≥0.416	1.35 (1.09–1.83)	0.041
P for likelihood test		0.019
**TyG-ABSI**		
All-cause mortality		
Model 1 Fitting model by standard linear regression	1.24 (1.14–1.34)	<0.001
Model 2 Fitting model by two-piecewise linear regression		
Inflection point	−0.604	
<-0.604	1.09 (0.68–1.75)	0.710
≥-0.604	1.26 (1.14–1.38)	<0.001
P for likelihood test		0.697
**CVD mortality**		
Model 1 Fitting model by standard linear regression	1.12 (0.97–1.29)	0.132
Model 2 Fitting model by two-piecewise linear regression		
Inflection point	1.688	
<1.688	1.11 (0.93–1.33)	0.244
≥1.688	0.41 (0.12–1.42)	0.160
P for likelihood test		0.778

Threshold analysis results were described using OR with 95%CI to quantify the non-linear associations. a: adjusted for age, sex, ethnicity, marital status, PIR, smoking status, drinking status, physical activity, education level, hypertension, and hyperlipidemia; CVD, cardiovascular disease; ABSI, A Body Shape Index; TyG-ABSI, Triglyceride Glucose - A Body Shape Index; HR, hazard ratios; CI, confidence interval; PIR, poverty-to-income ratio.

**Figure 2.**
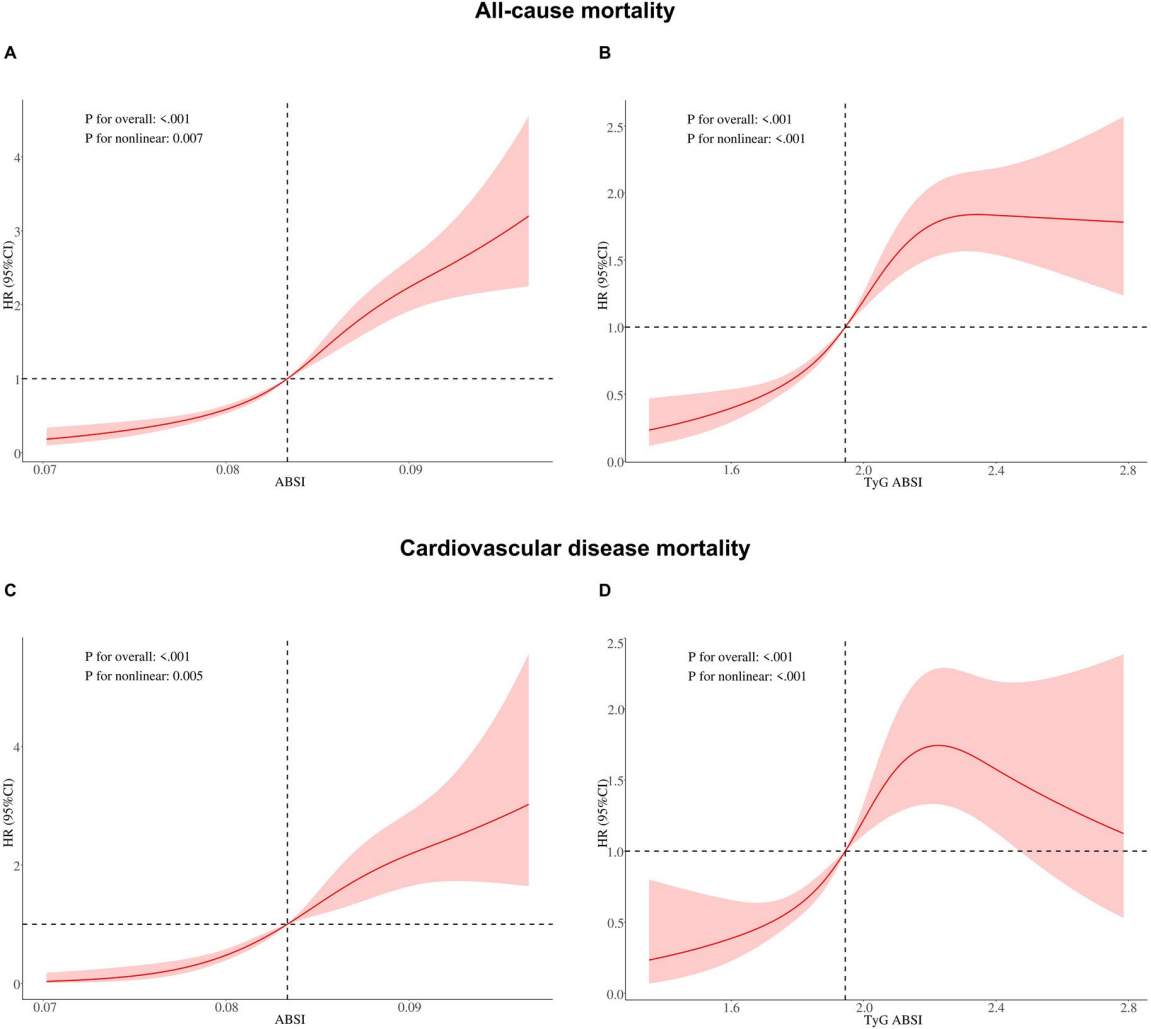
Dose-response associations of ABSI and TyG-ABSI with all-cause mortality and CVD mortality. (A) Association between ABSI and all-cause mortality. (B) Association between TyG-ABSI and all-cause mortality. (C) Association between ABSI and CVD mortality. (D) Association between TyG-ABSI and CVD mortality. HR, hazard ratios; CI, confidence interval; ABSI, A Body Shape Index; TyG-ABSI, Triglyceride Glucose - A Body Shape Index.

RCS were applied to examine nonlinear relationships and identify optimal thresholds for ABSI and TyG-ABSI z-scores with all-cause and CVD mortality (Figure 2). The analyses revealed significant nonlinear associations between these adiposity markers and both mortality outcomes (P for nonlinearity <0.001), with overall associations reaching statistical significance (*p* < 0.001). Subsequent threshold effect analysis identified significant inflection points for ABSI z-scores in relation to all-cause mortality (optimal threshold: 0.624 z-score units; P for likelihood ratio test <0.05) and CVD mortality (optimal threshold: 0.416 z-score units; P for likelihood ratio test <0.05), as detailed in Table 3. Notably, mortality risk exhibited a nonlinear steep increase when ABSI z-scores exceeded these threshold values, underscoring the critical role of ABSI in risk stratification for adverse clinical outcomes. In contrast, TyG-ABSI z-scores demonstrated no significant threshold effects for either outcome (P for likelihood ratio test >0.05).

### Prediction of all-cause and CVD mortality using ABSI and TyG-ABSI z-scores

In fully adjusted Cox models (Model 4 vs. Model 1), ABSI demonstrated robust mortality associations. For all-cause mortality, continuous ABSI (per 1-SD increase) showed 83% increased risk in Model 1 (HR = 1.83, 95%CI:1.66–2.01; *p* < 0.001) persisting at 16% in Model 4 (HR = 1.16,1.04–1.28; *p* = 0.006). Categorically (Q4 vs Q1), ABSI conferred 453% higher risk in Model 1 (HR = 5.53,4.20–7.28; *p* < 0.001) and 50% higher risk in Model 4 (HR = 1.50,1.09–2.05; *p* = 0.012). For CVD mortality, continuous ABSI showed 101% increased risk in Model 1 (HR = 2.01,1.78–2.28; *p* < 0.001) and 20% in Model 4 (HR = 1.20,1.05–1.38; *p* = 0.010); categorically, 1022% higher risk in Model 1 (HR = 11.22,6.31–19.97; *p* < 0.001) vs 140% in Model 4 (HR = 2.40,1.35–4.26; *p* = 0.003). The competing risk analysis using the Fine-Gray model for CVD mortality, accounting for non-CVD death, yielded results directionally consistent with the cause-specific Cox models (Table S4). ABSI remained significantly associated with CVD mortality in the fully adjusted model (sHR = 1.15, 95% CI 1.01–1.32, *p* = 0.040), while TyG-ABSI showed no significant association (sHR = 1.11, 95% CI 0.98–1.27, *p* = 0.100). TyG-ABSI associations attenuated: For all-cause mortality, continuous TyG-ABSI showed 46% increased risk in Model 1 (HR = 1.46,1.30–1.64; *p* < 0.001) vs 14% in Model 4 (HR = 1.14,1.03–1.26; *p* = 0.012), while categorical TyG-ABSI (Q4 vs Q1) showed 254% higher risk in Model 1 (HR = 3.54,2.67–4.69; *p* < 0.001) but non-significance in Model 4 (*p* = 0.152). For CVD mortality, continuous TyG-ABSI was non-significant in Model 4 (*p* = 0.650) despite Model 1 significance (HR = 1.41,1.23–1.62; *p* < 0.001), and categorical TyG-ABSI lost significance after Model 1 (Model 4 *p* = 0.955) (Table 4).

**Table 4.** Associations of ABSI z-scores and TyG-ABSI z-scores with mortality in CKD.

Variables	Model1		Model2		Model3		Model4	
	HR (95%CI)	*P*	HR (95%CI)	*P*	HR (95%CI)	*P*	HR (95%CI)	*P*
**All-cause mortality**								
ABSI	1.83 (1.66–2.01)	<.001	1.20 (1.09–1.33)	<.001	1.15 (1.04–1.27)	0.008	1.16 (1.04–1.28)	0.006
ABSIQ								
Q1	1.00 (Reference)		1.00 (Reference)		1.00 (Reference)		1.00 (Reference)	
Q2	1.98 (1.42–2.74)	<.001	1.27 (0.92–1.76)	0.150	1.26 (0.91–1.75)	0.165	1.27 (0.92–1.77)	0.151
Q3	3.30 (2.34–4.66)	<.001	1.55 (1.14–2.11)	0.005	1.47 (1.09–1.98)	0.012	1.47 (1.10–1.98)	0.010
Q4	5.53 (4.20–7.28)	<.001	1.62 (1.20–2.19)	0.002	1.47 (1.08–2.01)	0.014	1.50 (1.09–2.05)	0.012
TyG-ABSI	1.46 (1.30–1.64)	<.001	1.17 (1.07–1.28)	<.001	1.12 (1.02–1.24)	0.019	1.14 (1.03–1.26)	0.012
TyG-ABSIQ								
Q1	1.00 (Reference)		1.00 (Reference)		1.00 (Reference)		1.00 (Reference)	
Q2	1.68 (1.19–2.38)	0.004	1.06 (0.79–1.43)	0.675	1.07 (0.80–1.44)	0.638	1.09 (0.82–1.46)	0.539
Q3	2.24 (1.64–3.04)	<.001	1.08 (0.80–1.46)	0.597	1.06 (0.78–1.43)	0.729	1.08 (0.81–1.45)	0.589
Q4	3.54 (2.67–4.69)	<.001	1.31 (1.01–1.71)	0.049	1.21 (0.91–1.61)	0.184	1.22 (0.93–1.62)	0.152
**CVD mortality**								
ABSI	2.01 (1.78–2.28)	<.001	1.21 (1.06–1.38)	0.005	1.20 (1.04–1.37)	0.009	1.20 (1.05–1.38)	0.010
ABSIQ								
Q1	1.00 (Reference)		1.00 (Reference)		1.00 (Reference)		1.00 (Reference)	
Q2	4.43 (2.34–8.39)	<.001	2.49 (1.37–4.52)	0.003	2.55 (1.41–4.63)	0.002	2.62 (1.45–4.75)	0.001
Q3	6.71 (3.73–12.06)	<.001	2.48 (1.39–4.42)	0.002	2.48 (1.39–4.41)	0.002	2.58 (1.45–4.59)	0.001
Q4	11.22 (6.31–19.97)	<.001	2.37 (1.37–4.10)	0.002	2.29 (1.31–4.02)	0.004	2.40 (1.35–4.26)	0.003
TyG-ABSI	1.41 (1.23–1.62)	<.001	1.04 (0.89–1.22)	0.606	1.02 (0.87–1.20)	0.787	1.04 (0.88–1.24)	0.650
TyG-ABSIQ								
Q1	1.00 (Reference)		1.00 (Reference)		1.00 (Reference)		1.00 (Reference)	
Q2	1.81 (1.02–3.20)	0.042	0.99 (0.57–1.71)	0.959	1.00 (0.57–1.74)	0.989	1.05 (0.60–1.85)	0.857
Q3	2.60 (1.42–4.78)	0.002	1.04 (0.57–1.92)	0.892	1.03 (0.55–1.93)	0.921	1.09 (0.57–2.06)	0.796
Q4	3.46 (2.03–5.92)	<.001	1.01 (0.61–1.70)	0.957	0.97 (0.57–1.66)	0.911	1.02 (0.57–1.81)	0.955

Model 1 (crude adjustment); Model 2 (adjusted for age and sex); Model 3 (further adjusted for eth-nicity, education level, marital status, PIR, drinking status, smoking status, and physical activity level); and Model 4 (additionally adjusted for hypertension, and hyperlipidemia). CVD, cardiovascular disease; ABSI, A Body Shape Index; TyG-ABSI, Triglyceride Glucose - A Body Shape Index; HR, hazard ratios; CI, confidence interval; PIR, poverty-to-income ratio.

**Figure 3.**
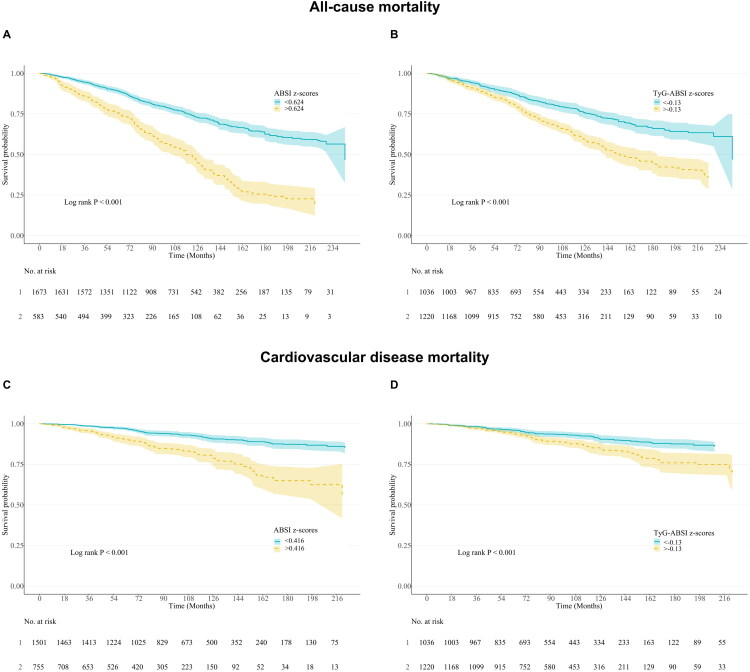
Kaplan-Meier survival curves for ABSI z-scores and TyG-ABSI z-scores with all-cause and CVD mortality. (A) Association between ABSI z-scores and all-cause mortality. (B) Association between TyG-ABSI z-scores and all-cause mortality. (C) Association between ABSI z-scores and CVD mortality. (D) Association between TyG-ABSI z-scores and CVD mortality. ABSI, A Body Shape Index; TyG-ABSI, Triglyceride Glucose - A Body Shape Index.

For ABSI, KM analysis was performed using the identified optimal ABSI z-score cutoffs for all-cause mortality (0.624) and CVD mortality (0.416). For TyG-ABSI, which exhibited no significant threshold effect, participants were stratified using the median TyG-ABSI z-score (−0.13). KM analysis demonstrated significantly lower survival probabilities in the higher ABSI and higher TyG-ABSI groups compared to their lower counterparts for both mortality outcomes (all log-rank *p*-values < 0.001; Figure 3).

### ROC analysis and subgroup analyses

**Figure 4.**
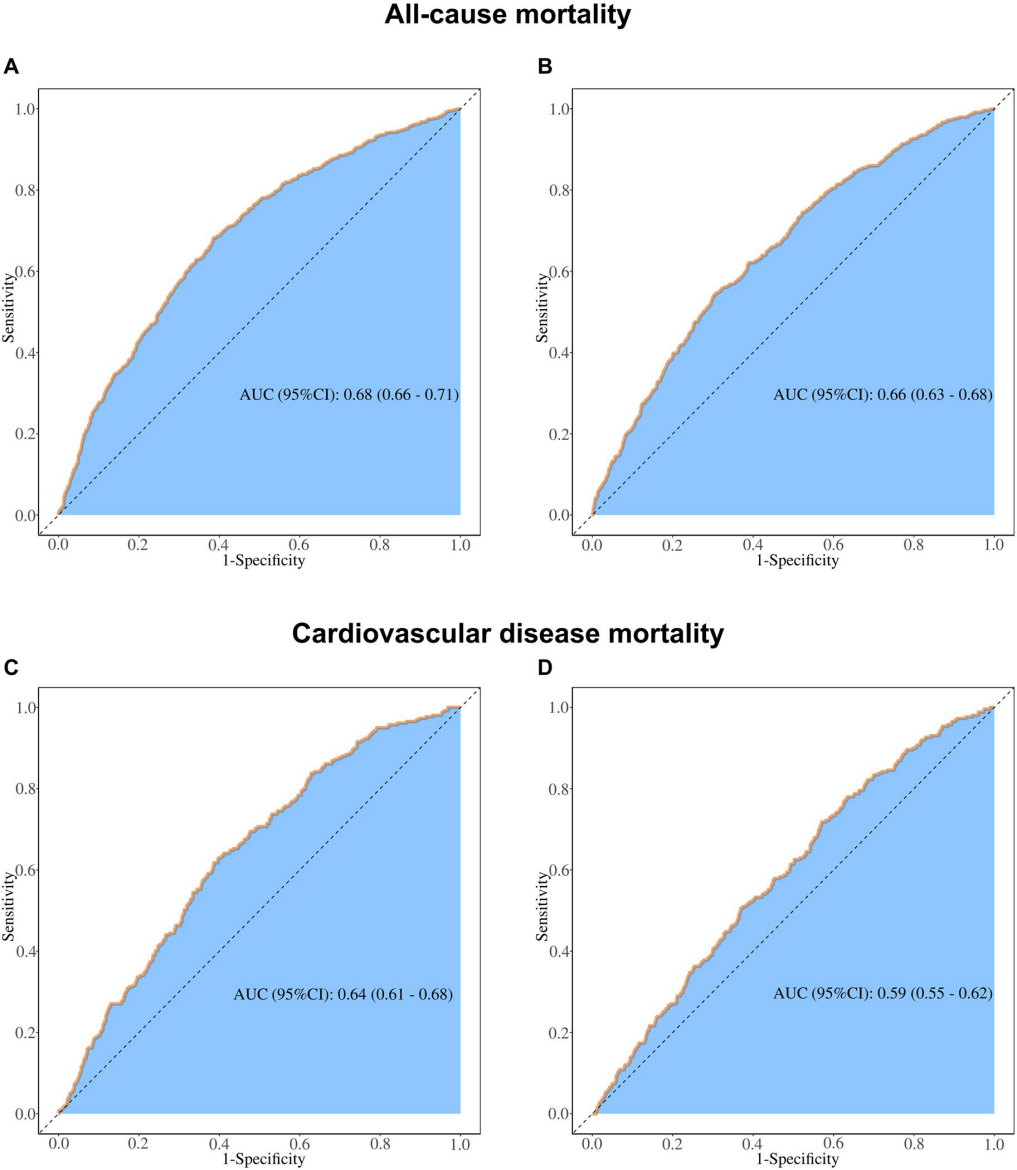
ROC curves of ABSI z-scores and TyG-ABSI z-scores for predicting all-cause and CVD mortality in patients with CKD, adjusted for Model 4 covariates. (A) Association between ABSI z-scores and all-cause mortality. (B) Association between TyG-ABSI z-scores and all-cause mortality. (C) Association between ABSI z-scores and CVD mortality. (D) Association between TyG-ABSI z-scores and CVD mortality. ABSI, A Body Shape Index; TyG-ABSI, Triglyceride Glucose - A Body Shape Index. AUC, area under the curve; CI, confidence interval.

ROC curve analysis revealed significant differences in the predictive performance of ABSI and TyG-ABSI z-scores for both all-cause and CVD mortality. The ABSI z-score demonstrated significant discriminative ability, with an AUC of 0.68 (95% CI: 0.66–0.71) for all-cause mortality (Figure 4(A)) and 0.64 (95% CI: 0.61–0.68) for CVD mortality (Figure 4(C)). In contrast, TyG-ABSI z-scores exhibited comparatively limited predictive power, yielding an AUC of 0.66 (95% CI: 0.63–0.68) for all-cause mortality (Figure 4(B)) and 0.59 (95% CI: 0.55–0.62) for CVD mortality (Figure 4(D)). To further assess model performance, we evaluated calibration and overall accuracy. The calibration curves for the Clinical model and models augmented with ABSI or TyG-ABSI showed excellent agreement between predicted and observed probabilities for both all-cause and CVD mortality at 3 and 5 years (Figure S2). The Brier scores were low and similar across all models (e.g. for 5-year all-cause mortality: Clinical model: 0.038975, Clinical + ABSI: 0.038767, Clinical + TyG-ABSI: 0.038843), indicating high overall accuracy (Table S5). The calibration slopes were close to 1.0, further confirming good calibration (Figure S3).

Furthermore, DCA was conducted to evaluate the clinical net benefit of using these indices for CKD risk stratification (Figure S1). The DCA for prevalent CKD demonstrated that models incorporating TyG-ABSI or ABSI provided a superior net benefit across a wide range of threshold probabilities compared to the strategies of intervening for all or no patients. The model with TyG-ABSI yielded the highest net benefit, indicating its stronger potential to inform clinical decisions.

**Figure 5.**
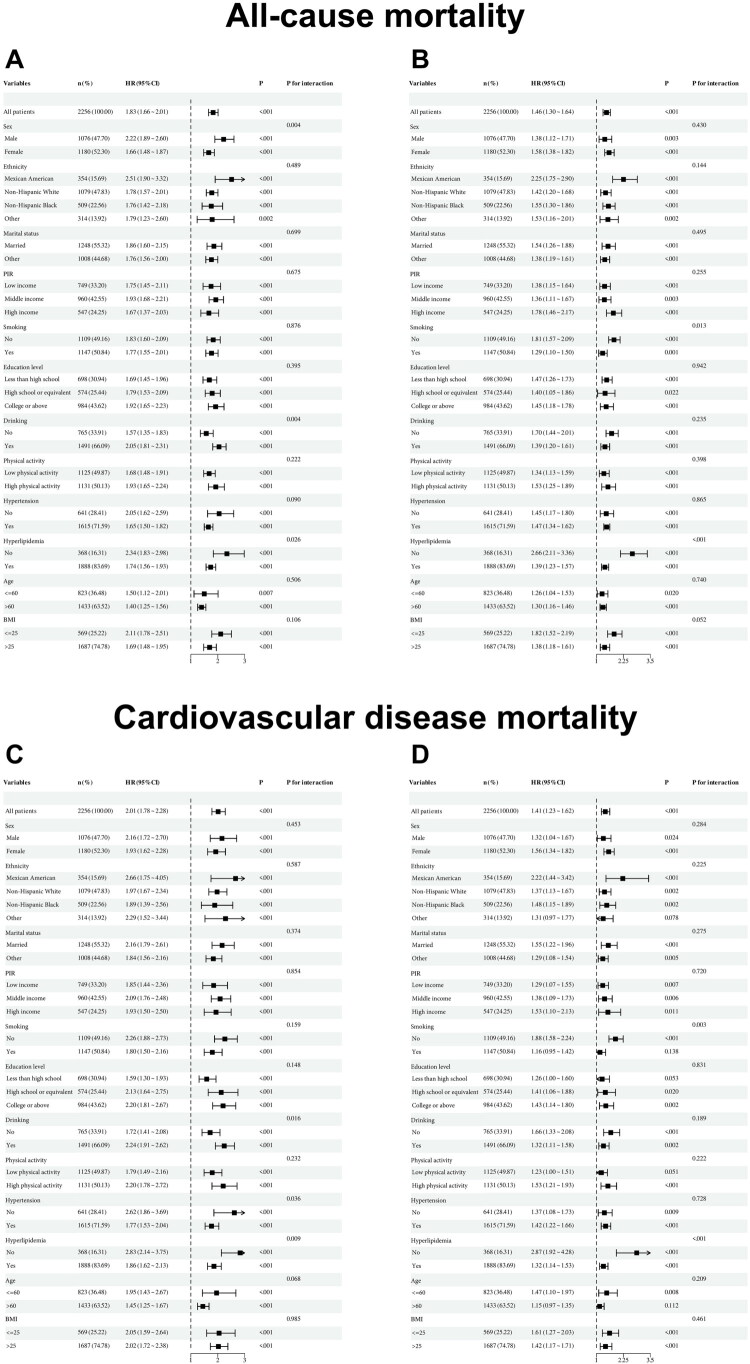
Subgroup analyses evaluating associations of ABSI z-scores and TyG-ABSI z-scores with all-cause mortality and CVD mortality. (A) Association between ABSI z-scores and all-cause mortality. (B) Association between TyG-ABSI z-scores and all-cause mortality. (C) Association between ABSI z-scores and CVD mortality. (D) Association between TyG-ABSI z-scores and CVD mortality. ABSI, A Body Shape Index; TyG-ABSI, Triglyceride Glucose - A Body Shape Index. HR, hazard ratios; CI, confidence interval; PIR, poverty-to-income ratio; BMI, body mass index.

Subgroup analyses and interaction tests were conducted for sex, age, BMI, race, educational level, marital status, alcohol consumption, smoking status, physical activity, PIR, hypertension, and dyslipidemia. For ABSI z-score, statistically significant associations (*p* < 0.05) were observed across all subgroups for both all-cause mortality (Figure 5(A)) and CVD mortality (Figure 5(C)). Significant effect modification was detected for all-cause mortality in subgroups defined by sex, alcohol consumption, and dyslipidemia status, and for CVD mortality in subgroups defined by alcohol consumption, hypertension, and dyslipidemia status (all p for interaction < 0.05). For TyG-ABSI z-score, statistically significant associations (*p* < 0.05) were observed across all subgroups for all-cause mortality (Figure 5(B)); however, for CVD mortality, associations were not significant in subgroups of smokers, individuals with lower educational attainment (below high school), low physical activity, and older age (>60 years) (Figure 5(D)). Significant effect modification was observed for both all-cause and CVD mortality in subgroups defined by smoking status and dyslipidemia status (all p for interaction< 0.05).

## Discussion

The NHANES data analysis presented in this investigation offers substantial insights into the differential clinical utility of anthropometric indices for CKD risk stratification and mortality prediction. This comprehensive evaluation demonstrates that while TyG-ABSI provides superior performance in identifying individuals at risk for CKD development, the standalone ABSI emerges as a more robust predictor of mortality in established CKD, particularly through its distinctive nonlinear associations with fatal outcomes [34]. This divergence in predictive capacity across the disease continuum reflects fundamental shifts in the pathophysiology of obesity-related renal pathology, wherein anatomical fat distribution gradually supersedes metabolic dysfunction as the primary driver of adverse events as CKD advances [35].

The enhanced discriminative capacity of TyG-ABSI for CKD prevalence represents a significant advancement over conventional obesity metrics. While BMI and WC provide general measures of overall and abdominal adiposity, and WHtR offers a simple correction for height, they remain primarily anatomical and are limited in their ability to directly quantify the underlying metabolic dysregulation that drives renal injury. Similarly, the TyG index alone is a robust surrogate of insulin resistance but lacks the anatomical component of central fat distribution. In contrast, TyG-ABSI uniquely captures two synergistic pathways in obesity-related nephropathy: the TyG component quantifies insulin resistance, which drives glomerular hyperfiltration, podocyte injury, and renal lipotoxicity through elevated free fatty acid flux and altered lipid metabolism; simultaneously [36], ABSI precisely measures the mechanical burden of visceral adiposity, which exerts direct physical compression on renal structures and secretes pro-inflammatory adipokines that promote fibrosis [37]. This multiplicative combination explains TyG-ABSI’s persistent association with CKD after comprehensive adjustment for cardiometabolic comorbidities, where traditional measures like body mass index lose predictive value. The retention of significant risk exclusively in the highest TyG-ABSI quartile following full covariate adjustment suggests a critical threshold effect—only severe combined metabolic-visceral derangement independently elevates CKD risk beyond conventional factors [38]. The superior net benefit of TyG-ABSI, as evidenced by the DCA, strengthens its potential for clinical application. This suggests that integrating TyG-ABSI could optimize decision-making for screening intensity, referral thresholds, and the selective use of confirmatory tests like albuminuria measurement, particularly in primary care settings where efficient risk stratification is paramount. This persistent association, even after adjustment for covariates that often confound BMI, underscores the integrated value of TyG-ABSI over its individual components or traditional measures.

This observation has profound clinical implications for early intervention strategies. Regarding the feasibility of integrating TyG-ABSI into primary care or EHR systems, we acknowledge that its manual calculation from three separate components (WC, TG, FBG) presents a practical barrier. However, this challenge is highly amenable to technological solutions. A key strategy would be the development of automated clinical decision support (CDS) tools within the EHR. These tools could be programmed to automatically calculate the TyG-ABSI score in the background whenever its three constituent variables are available from a patient’s record (e.g., following a routine health check or laboratory panel). The calculated value could then be displayed prominently on the clinician’s dashboard, potentially with a flag or alert for patients whose scores fall into the high-risk quartile (Q4), prompting consideration for further renal assessment (e.g., UACR testing) or more intensive management of cardiometabolic risk factors. In resource-limited settings without advanced EHR capabilities, the calculation could be simplified by incorporating it into the laboratory information system, with the TyG-ABSI value reported alongside the standard lipid and glucose results. Furthermore, patient-facing health apps could also be leveraged to calculate and track this index, fostering patient awareness and engagement in preventive health. Therefore, while the formula appears complex, its calculation is based on commonly and routinely measured clinical parameters. The primary hurdle is not the complexity of the formula itself, but the need for systematic data integration and presentation, which is a surmountable challenge in the era of digital health. By embedding TyG-ABSI into automated workflows, it can transition from a research metric to a practical, scalable tool for population-wide CKD risk stratification, effectively identifying high-risk phenotypes in the community before irreversible renal damage occurs.

Specifically, TyG-ABSI could be integrated into primary care screening protocols to identify individuals with preserved eGFR but high metabolic-visceral risk, who might otherwise be missed by current KDIGO criteria that primarily rely on eGFR and UACR [23]. This aligns with the KDIGO recommendation to identify and manage risk factors for CKD development. For patients with established CKD, the ABSI z-score thresholds can refine prognostication within the KDIGO risk categories (e.g. high and very high risk). A high ABSI value could signal the need for more aggressive management of cardiometabolic complications and closer monitoring, even if eGFR and UACR are stable, thereby personalizing the application of KDIGO’s treat-to-target goals.

As CKD progresses to advanced stages, the predictive hierarchy undergoes a decisive transition toward ABSI dominance. This shift highlights a key limitation of BMI, which is notoriously confounded by fluid overload and muscle wasting (sarcopenia) in advanced CKD, often leading to the ‘obesity paradox.’ WC and WHtR, while better than BMI for assessing central fat, are less effective than ABSI in normalizing for body shape and identifying the specific, high-risk visceral adiposity phenotype linked to inflammation and poor outcomes. The robustness of ABSI is further underscored by the competing risk analysis for CVD mortality, where ABSI remained a significant predictor even after accounting for non-CVD death, a common event in advanced CKD. The superior and nonlinear predictive power of ABSI for mortality, therefore, suggests it adds critical prognostic information not fully captured by these conventional anthropometric tools.

The identification of specific ABSI z-score thresholds—0.624 standard deviations for all-cause mortality and 0.416 for cardiovascular mortality—beyond which mortality risk escalates nonlinearly constitutes a major contribution to prognostication in nephrology [39]. This threshold phenomenon, conspicuously absent for TyG-ABSI, signals that body shape normalization becomes increasingly critical in late-stage disease. Beyond their statistical significance, these thresholds hold promise for refining risk stratification in clinical practice. For instance, an ABSI z-score persistently ≥ 0.624 in a patient with established CKD could serve as an indicator to intensify multifaceted management strategies. This may include escalating cardiorenal protective therapies (e.g., ensuring maximal tolerated doses of SGLT2 inhibitors and MRAs), implementing structured nutritional interventions targeting sarcopenic obesity, and enhancing physical rehabilitation programs to modify body composition. This approach aligns with the paradigm of precision medicine, where easily obtainable anthropometric data can help identify a high-risk phenotype that might benefit from earlier and more aggressive intervention, even in the absence of further decline in traditional metrics like eGFR. Integrating ABSI thresholds with existing KDIGO risk categories (based on eGFR and UACR) could create a more holistic risk profile, potentially triggering specialized consultations (e.g. with cardiology or nutrition) or increasing the frequency of clinical monitoring. Thus, ABSI is not proposed as a standalone tool but as a complementary, readily available biomarker that could enhance the personalization of care for patients navigating the advanced stages of CKD.

Several interconnected pathophysiological mechanisms explain this transition. A key factor is the prevalent sarcopenic obesity in advanced CKD, where progressive muscle wasting unmasks and accentuates the relative dominance of central adiposity—a phenomenon directly captured by ABSI’s focus on waist circumference normalized to height and weight. This body composition shift is compounded by the active redistribution of fat, with marked expansion of metabolically adverse visceral, perirenal, and renal sinus deposits, which are strongly correlated with ABSI and exert direct mechanical compression on renal structures, exacerbating interstitial hypertension and ischemic injury [40]. Concurrently, the uremic milieu fuels a state of chronic inflammation, characterized by elevated levels of cytokines such as IL-6 and TNF-α [41], which not only promotes muscle catabolism but also induces adipose tissue senescence and dysfunction. This inflamed, senescent visceral fat adopts a profibrotic secretory phenotype, releasing mediators like plasminogen activator inhibitor-1 and transforming growth factor-beta that directly drive renal fibrosis. Crucially, these anatomy-driven and inflammation-amplified pathways operate increasingly independently of the insulin resistance captured by the TyG index [42]. This shift in dominant pathophysiology is supported by recent evidence suggesting that the contribution of classical insulin resistance to renal and cardiovascular outcomes may be attenuated in advanced CKD, whereas the mechanical and inflammatory burden of visceral and ectopic fat deposits becomes paramount [43,44]. Thus, in advanced CKD, ABSI effectively integrates the net adverse impact of sarcopenic obesity, pathological fat redistribution, and chronic inflammation, evolving from a component of metabolic syndrome to the dominant independent anatomical risk entity in decompensated CKD, thereby surpassing the predictive value of TyG-ABSI. We therefore posit that the predictive superiority of ABSI in late-stage CKD may reflect this fundamental transition from a metabolically-driven to an anatomically- and inflammation-driven disease phenotype.

The superior mortality discrimination demonstrated by ABSI, with area under the curve values of 0.68 for all-cause mortality and 0.64 for cardiovascular mortality compared to TyG-ABSI’s 0.66 and 0.59 respectively, further validates this pathophysiological shift. The comprehensive performance evaluation, now including calibration and Brier scores, strengthens our conclusions. The excellent calibration and similar, low Brier scores across models indicate that while ABSI provides superior discrimination, all models are well-calibrated and accurate, reinforcing the reliability of the observed differential utility of the indices. This apparent paradox—where a composite index underperforms its anatomical constituent—stems from the progressive decoupling of metabolic and anatomical pathways as renal function deteriorates. Metabolic parameters like insulin sensitivity become increasingly confounded by malnutrition-inflammation complex, acid-base disturbances, anemia, and iatrogenic factors including insulin dosing and corticosteroid use. Simultaneously, visceral adipose tissue gains functional autonomy as an endocrine organ, secreting renalase and fibroblast growth factor-21 that directly modulate cardiac fibrosis and renal scarring [45]. The mechanical consequences of central obesity—including diaphragmatic stress, ventricular constraint, and venous congestion—also become more clinically consequential in fluid-overloaded CKD patients. Thus, while TyG-ABSI excels in capturing incipient risk in relatively preserved renal function, ABSI emerges as the dominant biomarker in the compromised renal environment characteristic of late-stage disease.

Subgroup analyses revealed clinically significant modifiers of these relationships that refine practical application. The stronger ABSI-mortality association observed in men compared to women likely reflects sex-dimorphic fat distribution patterns [46], where android obesity in males concentrates visceral burden around vital organs. The striking age interaction—with ABSI substantially outperforming TyG-ABSI in elderly populations—highlights its particular relevance in sarcopenic obesity [47], where progressive muscle loss exaggerates central adiposity without proportional weight gain. Hypertension dramatically amplified ABSI’s cardiovascular mortality hazard, with adjusted hazard ratios reaching 2.14 versus 1.29 in normotensive individuals, suggesting synergistic neurohormonal activation involving renal afferent signaling and sympathetic overactivity. Conversely, lifestyle factors significantly modulated these associations: regular physical activity attenuated ABSI-related mortality risk, implying that structured exercise may partially counteract the mechanical impacts of visceral adiposity through improved body composition and cardiorespiratory fitness. These critical nuances demand personalized index selection in clinical practice—favoring ABSI for elderly hypertensive males and TyG-ABSI for younger prediabetic cohorts—and underscore that contemporary obesity phenotyping must evolve beyond simplistic one-size-fits-all metrics toward precision-based approaches.

The implications of our findings extend well beyond nephrology. For cardiology, the robust association of ABSI with CVD mortality underscores its value as an anatomical risk marker that complements traditional cardiovascular risk scores. In endocrinology, TyG-ABSI’s strong link to CKD risk reinforces its potential for identifying patients with concurrent metabolic dysfunction and elevated renal disease susceptibility, a common clinical challenge in diabetes management. For geriatric medicine, ABSI’s superior performance in older CKD patients highlights its particular relevance in assessing sarcopenic obesity and predicting mortality in a population where conventional BMI is often misleading. Thus, the stage-specific application of these indices provides a unified framework for obesity phenotyping that can inform precision medicine across multiple clinical disciplines facing the challenges of cardiometabolic disease and aging populations.

The biological divergence between ABSI and metabolic indices in advanced CKD reflects activation of distinct pathological pathways. Unlike subcutaneous adipose depots, visceral fat in uremia exhibits profound mitochondrial dysfunction characterized by excessive fission [48], resulting in mitochondrial DNA release that activates renal Toll-like receptor 9 and NLRP3 inflammasomes. Crown-like structures within high-ABSI adipose tissue secrete C-C motif chemokine ligand 2 and resistin, recruiting galectin-3-producing macrophages that directly drive tubulointerstitial fibrosis through epithelial-mesenchymal transition. Crucially, these processes operate independently of insulin sensitivity. Simultaneously, renal sinus fat expansion—accurately captured by ABSI measurements—mechanically impedes venous and lymphatic drainage, elevating renal interstitial pressure and activating local renin-angiotensin-aldosterone systems [49]. This establishes a self-perpetuating cycle wherein fat-derived inflammation begets fibrosis, which subsequently entraps adipose tissue, further intensifying mechanical stress. These anatomy-driven pathways explain ABSI’s remarkable resilience as a mortality predictor even when conventional metabolic parameters lose prognostic relevance in late-stage disease.

Translating these insights into clinical practice necessitates concrete implementation strategies. TyG-ABSI should be incorporated into primary care CKD risk engines like the kidney failure risk equation to capture metabolically active obesity currently missed by conventional tools [50]. For nephrologists managing established CKD, ABSI thresholds should trigger intensive multimodal intervention: mechanical decompression *via* sodium-glucose cotransporter-2 inhibitors to reduce renal sinus fat volume [51]; senolytic therapies targeting adipose tissue senescence; and myokine modulation through structured exercise programs to elevate irisin’s anti-inflammatory effects. Serial ABSI measurements could effectively monitor visceral fat dynamics, complementing traditional trajectories of estimated glomerular filtration rate and albuminuria [52].

Public health initiatives should prioritize ABSI awareness, as its consistent association with mortality across BMI categories resolves the confounding “obesity paradox” that plagues conventional adiposity metrics. The robust and nonlinear association of ABSI with mortality in established CKD provides a potential key to deciphering the long-standing “obesity paradox,” wherein patients with higher BMI sometimes exhibit better survival. This paradox may largely stem from the inability of BMI to differentiate between protective muscle mass and detrimental adipose tissue, particularly in the context of sarcopenic obesity common in advanced CKD. In contrast, ABSI, by specifically normalizing waist circumference for height and weight, more accurately reflects the burden of central adiposity—a known driver of inflammation and cardiometabolic risk—while being less confounded by overall body size or muscle mass. Similarly, while waist circumference (WC) measures absolute abdominal size, it does not account for an individual’s overall body frame, a limitation ABSI is explicitly designed to overcome. Therefore, a high ABSI value likely identifies a phenotype of true “high-risk” obesity characterized by disproportionate visceral fat accumulation, irrespective of the BMI category. This refined discrimination helps explain why ABSI maintains a strong, graded relationship with mortality where BMI and WC often fail, thereby clarifying risk stratification and moving beyond the simplistic and often misleading interpretations generated by BMI alone.

This study introduces several key methodological innovations: First, the dual-index comparative framework (TyG-ABSI capturing metabolic-anatomical integration versus ABSI isolating body shape) addresses a critical gap in chronic kidney disease research by demonstrating stage-specific risk drivers. Second, restricted cubic spline analysis with maximum likelihood estimation precisely identified ABSI mortality thresholds (0.624 SD for all-cause; 0.416 SD for cardiovascular mortality), enabling binary clinical risk stratification. Third, our novel application of generalized variance inflation factors (VIF = GVIF^(1/(2 × Df))) for categorical covariates establishes a new standard for multicollinearity assessment in nutritional epidemiology. Finally, time-dependent ROC analysis with inverse probability weighting significantly enhances external validity of mortality prediction models.

Fourth, the discriminative accuracy of both ABSI and TyG-ABSI, as reflected by the AUC values (ranging from 0.59 to 0.68), is admittedly modest. This necessitates a careful consideration of their clinical actionability compared to conventional predictors such as BMI, waist circumference, or the rate of eGFR decline (eGFR slope). It is important to note that the primary contribution of this study lies not in proposing a single superior metric that supplants all others, but in demonstrating the differential utility of novel obesity phenotyping indices across the CKD continuum. While BMI and waist circumference provide a generalized assessment of adiposity, they are less effective in discriminating visceral fat mass and its specific metabolic and mechanical sequelae [6,7]. The eGFR slope is a powerful predictor of CKD progression but requires serial measurements over time. In this context, ABSI and TyG-ABSI offer distinct advantages: TyG-ABSI integrates central adiposity with underlying insulin resistance—a key pathophysiological driver—showing persistent association with CKD prevalence even after multivariable adjustment, suggesting its potential as a single, composite risk-screening tool. Conversely, in established CKD, ABSI’s robust, nonlinear association with mortality—and its ability to identify thresholds of sharply increasing risk—provides prognostic information that may be independent of and complementary to traditional risk factors, including baseline eGFR. Therefore, rather than judging their value solely by AUC, these indices may be most clinically actionable for specific tasks: TyG-ABSI for identifying high metabolic-visceral risk phenotypes in primary prevention, and ABSI for refining mortality risk stratification within nephrology clinics, potentially triggering intensified management in high-risk patients identified by the specified z-score thresholds.

Several study limitations warrant thoughtful consideration. The assessment of CKD was cross-sectional (prevalent CKD), which limits the ability to infer a temporal or causal relationship between the indices and the development of CKD. The observed associations for CKD risk should therefore be interpreted as identifying factors linked to the presence of the disease at a given timepoint. Although our analyses incorporated complex survey weights to enhance national representativeness and included extensive subgroup analyses that demonstrated consistent associations, we did not perform a formal sensitivity analysis directly comparing baseline characteristics between the included participants and those excluded due to missing data. While this is a common limitation in large database studies, it potentially limits our ability to fully assess the impact of selection bias on the observed associations. The single-timepoint assessment of indices cannot capture the clinical impact of weight cycling or dynamic metabolic changes; future research should incorporate longitudinal trajectory analyses. Despite rigorous adjustment for known confounders, residual confounding cannot be entirely excluded, necessitating Mendelian randomization studies to establish causal relationships. While the NHANES cohort includes diverse ethnic groups, its structure and specific population characteristics may limit the direct extrapolation of our results [53], particularly the identified ABSI z-score thresholds, to all global populations. The absence of advanced imaging correlation *via* computed tomography or dual-energy X-ray absorptiometry (DXA) limits direct anatomical validation of index components [54]. These gaps define essential research priorities: prospective interventional trials testing ABSI-guided therapeutic intensification; the development and validation of ethnicity-specific z-score equations or population-specific calibration of these indices; mechanistic studies exploring renal sinus fat relationships using magnetic resonance imaging; and comprehensive evaluation of ABSI’s role in dialysis and transplant outcomes.

## Conclusion

In conclusion, this investigation fundamentally reorients obesity phenotyping in renal medicine. The TyG-ABSI index represents a transformative tool for early CKD detection by integrating metabolic and anatomical dimensions of risk into a single clinically accessible metric. However, as chronic kidney disease advances toward end-stage manifestations, ABSI’s nonlinear threshold relationship with mortality reveals the escalating dominance of visceral fat mechanics over metabolic dysregulation in determining patient survival. This critical transition necessitates a staged approach to risk assessment: TyG-ABSI for population screening and primary prevention in community settings, with ABSI thresholds guiding prognostication and treatment personalization in established CKD managed by nephrology specialists. By transcending the limitations of conventional body mass index measurements and embracing body fat distribution as a dynamic risk entity, these complementary indices illuminate a pathway toward precision nephrology—where obesity management is strategically tailored not merely to total weight, but to the evolving interplay between body shape and metabolic health across the entire kidney disease spectrum.

## Supplementary Material

Supplementary File.docx

## Data Availability

The code and data generated during the study are available from the corresponding author upon reasonable request. The original data are publicly available on the NHANES website (https://wwwn.cdc.gov/nchs/nhanes/default.aspx).

## References

[1] GBD Chronic Kidney Disease Collaboration. Global, regional, and national burden of chronic kidney disease, 1990-2017: a systematic analysis for the Global Burden of Disease Study 2017. Lancet. 2020;395(10225):709–733. doi: 10.1016/S0140-6736(20)30045-3. 32061315 PMC7049905

[2] Kalantar-Zadeh K, Jafar TH, Nitsch D, et al. Chronic kidney disease. Lancet. 2021;398(10302):786–802. doi: 10.1016/S0140-6736(21)00519-5. 34175022

[3] Kovesdy CP, Furth SL, Zoccali C. World kidney day steering committee. Obesity and kidney disease: hidden consequences of the epidemic. J Nephrol. 2017;30:1–10. doi: 10.1007/s40620-017-0377-y. 28214961

[4] Stenvinkel P, Chertow GM, Devarajan P, et al. Chronic inflammation in chronic kidney disease progression: role of Nrf2. Kidney Int Rep. 2021;6(7):1775–1787. doi: 10.1016/j.ekir.2021.04.023. 34307974 PMC8258499

[5] Garofalo C, Borrelli S, Minutolo R, et al. A systematic review and meta-analysis suggests obesity predicts onset of chronic kidney disease in the general population. Kidney Int. 2017;91(5):1224–1235. doi: 10.1016/j.kint.2016.12.013. 28187985

[6] Ding C, Shi Y, Li J, et al. Association of weight-adjusted-waist index with all-cause and cardiovascular mortality in China: a prospective cohort study. Nutr Metab Cardiovasc Dis. 2022;32(5):1210–1217. doi: 10.1016/j.numecd.2022.01.033. 35277327

[7] Inagaki K, Tawada N, Takanashi M, et al. The association between body mass index and all-cause mortality in Japanese patients with incident hemodialysis. PLoS One. 2022;17(6):e0269849. doi: 10.1371/journal.pone.0269849. 35749459 PMC9231701

[8] Linge J, Borga M, West J, et al. Body composition profiling in the UK biobank imaging study. Obesity. 2018;26(11):1785–1795. doi: 10.1002/oby.22210. 29785727 PMC6220857

[9] Neeland IJ, Ross R, Després JP, et al. Visceral and ectopic fat, atherosclerosis, and cardiometabolic disease: a position statement. Lancet Diabetes Endocrinol. 2019;7(9):715–725. doi: 10.1016/S2213-8587(19)30084-1. 31301983

[10] Piché ME, Tchernof A, Després JP. Obesity phenotypes, diabetes, and cardiovascular diseases. Circ Res. 2020;126(11):1477–1500. doi: 10.1161/CIRCRESAHA.120.316101. 32437302

[11] Lee DH, Keum N, Hu FB, et al. Predicted lean body mass, fat mass, and all cause and cause specific mortality in men: prospective US cohort study. BMJ. 2018;362:k2575. doi: 10.1136/bmj.k2575. 29970408 PMC6028901

[12] Krakauer NY, Krakauer JC. A new body shape index predicts mortality hazard independently of body mass index. PLoS One. 2012;7(7):e39504. doi: 10.1371/journal.pone.0039504. 22815707 PMC3399847

[13] Navarro-González D, Sánchez-Íñigo L, Pastrana-Delgado J, et al. Triglyceride-glucose index (TyG index) in comparison with fasting plasma glucose improved diabetes prediction in patients with normal fasting glucose: the Vascular-Metabolic CUN cohort. Prev Med. 2016;86:99–105. doi: 10.1016/j.ypmed.2016.01.022. 26854766

[14] Guo VY, Yu EY, Wong CK, et al. Hypertriglyceridaemic-waist phenotype and risk of diabetes in people with impaired fasting glucose in primary care: a cohort study. Diabet Med. 2018;35(5):576–582. doi: 10.1111/dme.13601. 29438572

[15] Simental-Mendía LE, Rodríguez-Morán M, Guerrero-Romero F. The product of fasting glucose and triglycerides as surrogate for identifying insulin resistance in apparently healthy subjects. Metab Syndr Relat Disord. 2008;6(4):299–304. doi: 10.1089/met.2008.0034. 19067533

[16] Li Y, You A, Tomlinson B, et al. Insulin resistance surrogates predict hypertension plus hyperuricemia. J Diabetes Investig. 2021;12(11):2046–2053. doi: 10.1111/jdi.13573. PMC8565421 33982885

[17] Ji M, Zhang S, An R. Effectiveness of A Body Shape Index (ABSI) in predicting chronic diseases and mortality: a systematic review and meta-analysis. Obes Rev. 2018;19(5):737–759. doi: 10.1111/obr.12666. 29349876

[18] Wu L, Zhu W, Qiao Q, et al. Novel and traditional anthropometric indices for identifying metabolic syndrome in non-overweight/obese adults. Nutr Metab (Lond). 2021;18(1):3. doi: 10.1186/s12986-020-00536-x. 33407674 PMC7788902

[19] Liu XZ, Fan J, Pan SJ. METS-IR, a novel simple insulin resistance indexes, is associated with hypertension in normal-weight Chinese adults. J Clin Hypertens (Greenwich). 2019;21(8):1075–1081. doi: 10.1111/jch.13591. 31282098 PMC8030630

[20] Xia MF, Lin HD, Chen LY, et al. Association of visceral adiposity and its longitudinal increase with the risk of diabetes in Chinese adults: a prospective cohort study. Diabetes Metab Res Rev. 2018;34(7):e3048. doi: 10.1002/dmrr.3048. 30035847

[21] Carrero JJ, Hecking M, Chesnaye NC, et al. Sex and gender disparities in the epidemiology and outcomes of chronic kidney disease. Nat Rev Nephrol. 2018;14(3):151–164. doi: 10.1038/nrneph.2017.181. 29355169

[22] Tuttle KR, Alicic RZ, Duru OK, et al. Clinical characteristics of and risk factors for chronic kidney disease among adults and children: an analysis of the CURE-CKD registry. JAMA Netw Open. 2019;2(12):e1918169. doi: 10.1001/jamanetworkopen.2019.18169. 31860111 PMC6991307

[23] Torres VE, Ahn C, Barten TRM, et al. KDIGO 2025 clinical practice guideline for the evaluation, management, and treatment of autosomal dominant polycystic kidney disease (ADPKD): executive summary. Kidney Int. 2025;107(2):e234–e254. doi: 10.1016/j.kint.2024.07.010. 39848746

[24] Gansevoort RT, Correa-Rotter R, Hemmelgarn BR, et al. Chronic kidney disease and cardiovascular risk: epidemiology, mechanisms, and prevention. Lancet. 2013;382(9889):339–352. doi: 10.1016/S0140-6736(13)60595-4. 23727170

[25] Chen TC, Clark J, Riddles MK, et al. National health and nutrition examination survey, 2015-2018: sample design and estimation procedures. Vital Health Stat 2. 2020;(184):1–35. 33663649

[26] Zipf G, Chiappa M, Porter KS, et al. National health and nutrition examination survey: plan and operations, 1999-2010. Vital Health Stat 1. 2013;(56):1–37. 25078429

[27] Mirel LB, El Bural Félix S, Zhang C, et al. Comparative analysis of the national health interview survey public-use and restricted-use linked mortality files. Natl Health Stat Report. 2020;(143):1–32. 32600514

[28] Qiu S, Zhu J, Yuan M, et al. Non-linear correlation between the ratio of high-density lipoprotein cholesterol to C-reactive protein and all-cause mortality in adults: an extensive study based on nationwide data. Popul Health Metr. 2025;23(1):32. doi: 10.1186/s12963-025-00396-8. 40597306 PMC12211658

[29] Collins GS, Reitsma JB, Altman DG, et al. Transparent reporting of a multivariable prediction model for individual prognosis or diagnosis (TRIPOD): the TRIPOD statement. Ann Intern Med. 2015;162(1):55–63. doi: 10.7326/M14-0697. 25560714

[30] Shi J. Ambient ammonium exposure is associated with physical dysfunction in older adults in China. Sci Rep. 2025;15(1):19162. doi: 10.1038/s41598-025-04289-6. 40450064 PMC12126504

[31] Bao Y, Wang L, Du C, et al. Association between systemic immune inflammation index and cognitive impairment after acute ischemic stroke. Brain Sci. 2023;13(3):464. doi: 10.3390/brainsci13030464. 36979274 PMC10046597

[32] Kashiwazaki D, Chida K, Yoshida K, et al. Clinical features, radiological findings, and outcome in patients with symptomatic mild carotid stenosis: a MUSIC study. J Neurosurg. 2025;143(1):285–295. doi: 10.3171/2024.10.JNS241185. 40601988

[33] Blanche P, Dartigues JF, Jacqmin-Gadda H. Estimating and comparing time-dependent areas under receiver operating characteristic curves for censored event times with competing risks. Stat Med. 2013;32(30):5381–5397. doi: 10.1002/sim.5958. 24027076

[34] Cheng YJ, Chen ZG, Wu SH, et al. Body mass index trajectories during mid to late life and risks of mortality and cardiovascular outcomes: results from four prospective cohorts. EClinicalMedicine. 2021;33:100790. doi: 10.1016/j.eclinm.2021.100790. 33778436 PMC7985466

[35] Foster MC, Hwang SJ, Porter SA, et al. Fatty kidney, hypertension, and chronic kidney disease: the Framingham Heart Study. Hypertension. 2011;58(5):784–790. doi: 10.1161/HYPERTENSIONAHA.111.175315. 21931075 PMC3204377

[36] Shi M, Ma L, Fu P. Role of fatty acid binding protein 4 (FABP4) in kidney disease. Curr Med Chem. 2020;27(22):3657–3664. doi: 10.2174/0929867325666181008154622. 30306857

[37] Kang P, Sun B, Hao J, et al. Perirenal fat and chronic kidney disease: a systematic review and meta-analysis. Kidney Blood Press Res. 2025;50(1):240–248. doi: 10.1159/000543989. 40058346

[38] Xu X, Zhao Y, Zhao Z, et al. Correlation of visceral adiposity index with chronic kidney disease in the People’s Republic of China: to rediscover the new clinical potential of an old indicator for visceral obesity. Ther Clin Risk Manag. 2016;12:489–494. doi: 10.2147/TCRM.S96340. 27099507 PMC4820234

[39] Nunnari A, Di Girolamo FG, Teraž K, et al. The abdominal adiposity index (a body shape index) predicts 10-year all-cause mortality in elderly active non-obese subjects. J Clin Med. 2024;13(20):6155. doi: 10.3390/jcm13206155. 39458105 PMC11508734

[40] Fang Y, Xu Y, Yang Y, et al. The relationship between perirenal fat thickness and reduced glomerular filtration rate in patients with type 2 diabetes. J Diabetes Res. 2020;2020:6076145–6076147. doi: 10.1155/2020/6076145. 32685560 PMC7341433

[41] Tan H, Xu J, Liu Y. Ageing, cellular senescence and chronic kidney disease: experimental evidence. Curr Opin Nephrol Hypertens. 2022;31(3):235–243. doi: 10.1097/MNH.0000000000000782. 35142744 PMC9035037

[42] Gastaldelli A, Gaggini M, DeFronzo RA. Role of adipose tissue insulin resistance in the natural history of type 2 diabetes: results from the san antonio metabolism study. Diabetes. 2017;66(4):815–822. doi: 10.2337/db16-1167. 28052966

[43] Scurt FG, Ganz MJ, Herzog C, et al. Association of metabolic syndrome and chronic kidney disease. Obes Rev. 2024;25(1):e13649. doi: 10.1111/obr.13649. 37783465

[44] Qin Z, Chen X, Sun J, et al. The association between visceral adiposity index and decreased renal function: A population-based study. Front Nutr. 2023;10:1076301. doi: 10.3389/fnut.2023.1076301. 36969806 PMC10036366

[45] Suassuna PGA, de Paula RB, Sanders-Pinheiro H, et al. Fibroblast growth factor 21 in chronic kidney disease. J Nephrol. 2019;32(3):365–377. doi: 10.1007/s40620-018-0550-y. 30430412 PMC6483847

[46] Peters SAE, Bots SH, Woodward M. Sex differences in the association between measures of general and central adiposity and the risk of myocardial infarction: results from the UK biobank. J Am Heart Assoc. 2018;7(5):e008507. doi: 10.1161/JAHA.117.008507. 29490971 PMC5866342

[47] Liu P, Hao Q, Hai S, et al. Sarcopenia as a predictor of all-cause mortality among community-dwelling older people: a systematic review and meta-analysis. Maturitas. 2017;103:16–22. doi: 10.1016/j.maturitas.2017.04.007. 28778327

[48] Eirin A, Thaler R, Glasstetter LM, et al. Obesity-driven mitochondrial dysfunction in human adipose tissue-derived mesenchymal stem/stromal cells involves epigenetic changes. Cell Death Dis. 2024;15(6):387. doi: 10.1038/s41419-024-06774-8. 38824145 PMC11144257

[49] Chughtai HL, Morgan TM, Rocco M, et al. Renal sinus fat and poor blood pressure control in middle-aged and elderly individuals at risk for cardiovascular events. Hypertension. 2010;56(5):901–906. doi: 10.1161/HYPERTENSIONAHA.110.157370. 20837881 PMC3634339

[50] Tangri N, Grams ME, Levey AS, et al. Multinational assessment of accuracy of equations for predicting risk of kidney failure: a meta-analysis. JAMA. 2016;315(2):164–174. doi: 10.1001/jama.2015.18202. 26757465 PMC4752167

[51] Heerspink HJL, Kosiborod M, Inzucchi SE, et al. Renoprotective effects of sodium-glucose cotransporter-2 inhibitors. Kidney Int. 2018;94(1):26–39. doi: 10.1016/j.kint.2017.12.027. 29735306

[52] Li CI, Liu CS, Lin CH, et al. Association of body indices and risk of mortality in patients with type 2 diabetes. BMJ Open Diabetes Res Care. 2023;11(4):e003474. doi: 10.1136/bmjdrc-2023-003474. PMC10445358 37607771

[53] Wells JC, Saunders MA, Lea AS, et al. Beyond Bergmann’s rule: global variability in human body composition is associated with annual average precipitation and annual temperature volatility. Am J Phys Anthropol. 2019;170(1):75–87. doi: 10.1002/ajpa.23890. 31318051

[54] Lee JJ, Pedley A, Hoffmann U, et al. Cross-sectional associations of computed tomography (ct)-derived adipose tissue density and adipokines: the Framingham heart study. J Am Heart Assoc. 2016;5(3):e002545. doi: 10.1161/JAHA.115.002545. 26927600 PMC4943240

