# Supplementary material for: Stage-specific utility of obesity indices across the chronic kidney disease continuum
Source: Ren Fail. 2026 Mar 25;48(1):2646000. doi: 10.1080/0886022X.2026.2646000 (PMC13021019; doi:10.1080/0886022X.2026.2646000)
Supplement: Supplementary File.docx [file IRNF_A_2646000_SM0144.docx]

**Comparative Performance of A Body Shape Index and Triglyceride Glucose–A Body Shape Index for Chronic Kidney Disease Risk and Mortality Outcomes: Differential Utility Across Disease Continuum**

Zhengyang Zhu^a, *^, Kejun Ren^b, **^, Xiaowei Duan^a^, Xulei Hu^a^, Yong Lv^b^, Dong Wang^b^, Jin Hua^b^, Lei Zhang^b^, Yiping Wang^b^

^a^ First Clinical Medical College, Anhui University of Chinese Medicine, Hefei, Anhui Province, China

^b^ First Affiliated Hospital, Anhui University of Chinese Medicine, Hefei, Anhui Province, China.

SUPPLEMENTARY MATERIAL

Supplementary Methods

**Methods S1:** **Decision Curve Analysis for Evaluating and Comparing Clinical Utility (Net Benefit)**

DCA was performed to quantify the clinical net benefit of prediction models for prevalent CKD. Five distinct clinical strategies were evaluated and compared: 1) "Treat All", where all individuals are considered positive for CKD; 2) "Treat None", where no individuals are considered positive; 3) a "Clinical Model" based on a logistic regression model including sex, age, race, education level, marital status, PIR, alcohol consumption, smoking status, physical activity level, hypertension, and hyperlipidemia; 4) a "Clinical + ABSI z-score" model that augmented the Clinical Model with the standardized A Body Shape Index; and 5) a "Clinical + TyG-ABSI z-score" model that augmented the Clinical Model with the standardized Triglyceride Glucose-ABSI. The net benefit for each strategy was calculated across a continuous range of threshold probabilities (from 0% to 100%), which represent the minimum probability of CKD at which a clinician would deem an intervention (e.g., further testing or referral) justified. The net benefit was computed as the proportion of true positives minus the proportion of false positives, weighted by the odds of the threshold probability, thus integrating the trade-offs between clinical benefits and harms. The relative superiority of the strategies was determined by comparing their net benefits across the spectrum of threshold probabilities, with a higher net benefit indicating a more clinically useful model for informing decisions regarding screening intensity, referral thresholds, or albuminuria testing.

**Methods S2:** **Competing Risk Analysis for Cardiovascular Disease Mortality**

The association of ABSI and TyG-ABSI with CVD mortality was evaluated using competing risks regression analysis to account for non-CVD mortality as a competing event. A competing event is an alternative outcome that precludes the occurrence of the primary event of interest (CVD mortality). In this framework, standard Cox regression may overestimate the cumulative incidence of the primary event. We therefore employed the Fine-Gray proportional subdistribution hazards model, which directly models the hazard of the cumulative incidence function (often termed the subdistribution hazard). This approach allows for the estimation of subdistribution hazard ratios (sHR), which quantify the effect of a covariate on the risk of the primary event in the presence of competing events. The analysis was performed using the crr function from the cmprsk package in R. The same four hierarchical adjustment models as in the primary Cox analysis were fitted. The results, presented as sHR with 95% confidence intervals, are shown alongside the cause-specific hazard ratios (csHR) from the standard Cox models in Supplementary Table S4 for comparative purposes.

**Methods S3: Competing Risk Analysis for Cardiovascular Disease Mortality**

Model calibration was assessed using calibration curves, which plot the predicted probabilities of an event against the observed frequencies. A perfectly calibrated model would follow the 45-degree line. We generated calibration curves for the Clinical model (adjusted for sex, age, race, education level, marital status, PIR, alcohol consumption, smoking status, physical activity level, hypertension, and hyperlipidemia), the Clinical + ABSI z-score model, and the Clinical + TyG-ABSI z-score model. Calibration was evaluated at 3-year and 5-year time horizons for both all-cause and CVD mortality outcomes. The overall accuracy of the predictions was quantified using the Brier score, which measures the mean squared difference between the predicted probabilities and the actual outcomes; a lower Brier score indicates better accuracy. The calibration slope was also computed, with a value of 1 indicating perfect calibration. These analyses were conducted using the riskRegressionpackage in R. The calibration curves are presented in Supplementary Figure S2, and a comparative summary of the calibration slopes and Brier scores is shown in Supplementary Figure S3. We performed overall calibration rather than stratification by CKD stage due to the limited sample size within each stage, which would lead to unstable estimates and reduce the reliability of stage-specific calibration plots.

Supplementary Table

**Table S1.** **Multivariable model covariates selected by GVIF Criteria.**

| **Final Covariate^a^** | **GVIF** | **Df** | **VIF^b^** |
| --- | --- | --- | --- |
| Sex | 1.135 | 1 | 1.065 |
| Ethnicity | 1.357 | 3 | 1.052 |
| Marital status | 1.086 | 1 | 1.042 |
| PIR | 1.322 | 2 | 1.072 |
| Smoking | 1.173 | 1 | 1.083 |
| Education level | 1.349 | 2 | 1.078 |
| Drinking | 1.192 | 1 | 1.092 |
| Physical activity | 1.074 | 1 | 1.036 |
| Hypertension | 1.352 | 1 | 1.163 |
| Hyperlipidemia | 1.137 | 1 | 1.066 |
| Age | 1.749 | 1 | 1.322 |

**Note:** a: covariates retained after excluding variables with GVIF^(1/(2*Df)) ≥5. b: VIF = GVIF^(1/(2*Df)). GVIF, generalized variance inflation factor; BMI, body mass index; Df, degrees of freedom; VIF, variance inflation factor.

**Table S2.** **Baseline Characteristics Stratified by All-Cause Mortality Status in CKD Patients.**

| **Variable** | **Total**  **(n = 2256)** | **CKD patients alive (n=1480)** | **CKD patients dead (n=776)** | **Statistic** | ***P*** |
| --- | --- | --- | --- | --- | --- |
|  |  |  |  |  |  |
| SCR, Mean (SD) | 93.87 (1.58) | 86.55 (1.29) | 110.08 (4.12) | t=5.41 | <.001 |
| Weight, Mean (SD) | 83.29 (0.76) | 84.56 (0.99) | 80.48 (1.12) | t=-2.70 | 0.008 |
| Height, Mean (SD) | 166.55 (0.29) | 166.75 (0.39) | 166.10 (0.41) | t=-1.13 | 0.259 |
| BMI, Mean (SD) | 29.79 (0.24) | 30.17 (0.30) | 28.93 (0.33) | t=-2.84 | 0.005 |
| Age, Mean (SD) | 58.45 (0.60) | 53.13 (0.74) | 70.21 (0.58) | t=18.56 | <.001 |
| UACR, Mean (SD) | 187.47 (14.84) | 142.48 (13.21) | 287.02 (38.71) | t=3.48 | <.001 |
| eGFR, Mean (SD) | 81.84 (0.98) | 88.42 (1.29) | 67.29 (1.54) | t=-9.74 | <.001 |
| ABSI, Mean (SD) | 0.0832 (0.0001) | 0.0823 (0.0002) | 0.0853 (0.0002) | t=10.91 | <.001 |
| Tyg ABSI, Mean (SD) | 1.9576 (0.0066) | 1.9157 (0.0083) | 2.0501 (0.0110) | t=9.25 | <.001 |
| Sex, n (%) |  |  |  | χ²=17.99 | 0.003 |
| Male | 1076 (43.44) | 641 (40.46) | 435 (50.02) |  |  |
| Female | 1180 (56.56) | 839 (59.54) | 341 (49.98) |  |  |
| Ethnicity, n (%) |  |  |  | χ²=47.50 | <.001 |
| Mexican American | 354 (7.51) | 270 (9.18) | 84 (3.82) |  |  |
| Non-Hispanic White | 1079 (69.30) | 594 (64.98) | 485 (78.86) |  |  |
| Non-Hispanic Black | 509 (12.28) | 369 (13.47) | 140 (9.65) |  |  |
| Other | 314 (10.91) | 247 (12.37) | 67 (7.67) |  |  |
| Marital status, n (%) |  |  |  | χ²=20.33 | 0.003 |
| Married | 1248 (59.13) | 866 (62.26) | 382 (52.18) |  |  |
| Other (widowed, divorced,  separated, never married,  living with a partner) | 1008 (40.87) | 614 (37.74) | 394 (47.82) |  |  |
| PIR, n (%) |  |  |  | χ²=25.09 | 0.002 |
| Low income | 749 (25.04) | 488 (24.98) | 261 (25.19) |  |  |
| Middle income | 960 (41.29) | 599 (38.27) | 361 (47.96) |  |  |
| High income | 547 (33.67) | 393 (36.75) | 154 (26.85) |  |  |
| Smoking, n (%) |  |  |  | χ²=18.71 | <.001 |
| No | 1109 (48.53) | 786 (51.59) | 323 (41.76) |  |  |
| Yes | 1147 (51.47) | 694 (48.41) | 453 (58.24) |  |  |
| Education level, n (%) |  |  |  | χ²=26.27 | <.001 |
| Less than high school | 698 (21.80) | 434 (20.65) | 264 (24.34) |  |  |
| high school or equivalent | 574 (26.45) | 354 (24.04) | 220 (31.79) |  |  |
| college or above | 984 (51.75) | 692 (55.31) | 292 (43.87) |  |  |
| Drinking, n (%) |  |  |  | χ²=3.87 | 0.130 |
| No | 765 (31.30) | 503 (30.01) | 262 (34.16) |  |  |
| Yes | 1491 (68.70) | 977 (69.99) | 514 (65.84) |  |  |
| Physical activity, n (%) |  |  |  | χ²=24.73 | <.001 |
| Low physical activity | 1125 (45.58) | 681 (42.08) | 444 (53.34) |  |  |
| High physical activity | 1131 (54.42) | 799 (57.92) | 332 (46.66) |  |  |
| Hypertension, n (%) |  |  |  | χ²=149.20 | <.001 |
| No | 641 (35.30) | 511 (43.56) | 130 (17.02) |  |  |
| Yes | 1615 (64.70) | 969 (56.44) | 646 (82.98) |  |  |
| Hyperlipidemia, n (%) |  |  |  | χ²=13.54 | 0.003 |
| No | 368 (16.95) | 258 (18.91) | 110 (12.63) |  |  |
| Yes | 1888 (83.05) | 1222 (81.09) | 666 (87.37) |  |  |

**Note:** All estimates accounted for complex survey designs. NHANES, National Health and Nutrition Examination Survey; CKD, chronic kidney disease; BMI, body mass index; ABSI, a body shape index. TyG-ABSI; triglyceride glucose- a body shape index; PIR, poverty-to-income ratio; eGFR, estimated glomerular filtration rate; UACR, urinary albumin-to-creatinine ratio; SCR, serum creatinine; SD, standard deviation; t: t-test; χ²: Chi-square test.

**Table S3.** **Baseline Characteristics Stratified by CVD Mortality Status in CKD Patients.**

| **Variable** | **Total**  **(n = 2256)** | **CKD patients alive (n=1997)** | **CKD patients dead (n=259)** | **Statistic** | ***P*** |
| --- | --- | --- | --- | --- | --- |
|  |  |  |  |  |  |
| SCR, Mean (SD) | 93.87 (1.58) | 92.20 (1.64) | 109.04 (4.72) | t=3.37 | <.001 |
| Weight, Mean (SD) | 83.29 (0.76) | 83.49 (0.82) | 81.49 (1.82) | t=-0.99 | 0.324 |
| Height, Mean (SD) | 166.55 (0.29) | 166.65 (0.31) | 165.67 (0.81) | t=-1.13 | 0.262 |
| BMI, Mean (SD) | 29.79 (0.24) | 29.83 (0.26) | 29.44 (0.50) | t=-0.68 | 0.500 |
| Age, Mean (SD) | 58.45 (0.60) | 56.87 (0.66) | 72.69 (0.79) | t=14.99 | <.001 |
| UACR, Mean (SD) | 187.47 (14.84) | 171.63 (13.42) | 330.91 (83.10) | t=1.90 | 0.059 |
| eGFR, Mean (SD) | 81.84 (0.98) | 83.73 (1.06) | 64.70 (1.75) | t=-9.03 | <.001 |
| ABSI, Mean (SD) | 0.08 (0.00) | 0.08 (0.00) | 0.09 (0.00) | t=8.82 | <.001 |
| Tyg ABSI, Mean (SD) | 1.96 (0.01) | 1.95 (0.01) | 2.04 (0.02) | t=4.75 | <.001 |
| Sex, n (%) |  |  |  | χ²=15.28 | <.001 |
| Male | 1076 (43.44) | 923 (42.08) | 153 (55.71) |  |  |
| Female | 1180 (56.56) | 1074 (57.92) | 106 (44.29) |  |  |
| Ethnicity, n (%) |  |  |  | χ²=10.80 | 0.005 |
| Mexican American | 354 (7.51) | 329 (7.84) | 25 (4.56) |  |  |
| Non-Hispanic White | 1079 (69.30) | 919 (68.30) | 160 (78.30) |  |  |
| Non-Hispanic Black | 509 (12.28) | 455 (12.46) | 54 (10.65) |  |  |
| Other | 314 (10.91) | 294 (11.40) | 20 (6.49) |  |  |
| Marital status, n (%) |  |  |  | χ²=2.25 | 0.233 |
| Married | 1248 (59.13) | 1120 (59.64) | 128 (54.46) |  |  |
| Other (widowed, divorced,  separated, never married,  living with a partner) | 1008 (40.87) | 877 (40.36) | 131 (45.54) |  |  |
| PIR, n (%) |  |  |  | χ²=13.58 | 0.007 |
| Low income | 749 (25.04) | 675 (25.69) | 74 (19.18) |  |  |
| Middle income | 960 (41.29) | 829 (40.03) | 131 (52.71) |  |  |
| High income | 547 (33.67) | 493 (34.28) | 54 (28.11) |  |  |
| Smoking, n (%) |  |  |  | χ²=1.20 | 0.325 |
| No | 1109 (48.53) | 990 (48.91) | 119 (45.07) |  |  |
| Yes | 1147 (51.47) | 1007 (51.09) | 140 (54.93) |  |  |
| Education level, n (%) |  |  |  | χ²=3.71 | 0.304 |
| Less than high school | 698 (21.80) | 622 (21.71) | 76 (22.59) |  |  |
| high school or equivalent | 574 (26.45) | 497 (25.92) | 77 (31.23) |  |  |
| college or above | 984 (51.75) | 878 (52.36) | 106 (46.18) |  |  |
| Drinking, n (%) |  |  |  | χ²=1.92 | 0.177 |
| No | 765 (31.30) | 677 (30.85) | 88 (35.37) |  |  |
| Yes | 1491 (68.70) | 1320 (69.15) | 171 (64.63) |  |  |
| Physical activity, n (%) |  |  |  | χ²=6.16 | 0.031 |
| Low physical activity | 1125 (45.58) | 982 (44.72) | 143 (53.41) |  |  |
| High physical activity | 1131 (54.42) | 1015 (55.28) | 116 (46.59) |  |  |
| Hypertension, n (%) |  |  |  | χ²=43.41 | <.001 |
| No | 641 (35.30) | 597 (37.51) | 44 (15.35) |  |  |
| Yes | 1615 (64.70) | 1400 (62.49) | 215 (84.65) |  |  |
| Hyperlipidemia, n (%) |  |  |  | χ²=0.29 | 0.646 |
| No | 368 (16.95) | 328 (17.09) | 40 (15.68) |  |  |
| Yes | 1888 (83.05) | 1669 (82.91) | 219 (84.32) |  |  |

**Note:** All estimates accounted for complex survey designs. NHANES, National Health and Nutrition Examination Survey; CKD, chronic kidney disease; BMI, body mass index; ABSI, a body shape index. TyG-ABSI; triglyceride glucose- a body shape index; PIR, poverty-to-income ratio; eGFR, estimated glomerular filtration rate; UACR, urinary albumin-to-creatinine ratio; SCR, serum creatinine; SD, standard deviation; t: t-test; χ²: Chi-square test.

**Table S4.** **Comparisons of Cox Proportional Hazards and Fine-Gray Competing Risk Regression Models for Associations of ABSI and TyG-ABSI with CVD Mortality.**

| **Variables** | **Model1** | |  | **Model2** | |  | **Model3** | |  | **Model4** | |
| --- | --- | --- | --- | --- | --- | --- | --- | --- | --- | --- | --- |
|  | **HR (95%CI)** | ***P*** |  | **HR (95%CI)** | ***P*** |  | **HR (95%CI)** | ***P*** |  | **HR (95%CI)** | ***P*** |
| Fine-Gray |  |  |  |  |  |  |  |  |  |  |  |
| ABSI | 1.59 (1.43–1.77) | <.001 |  | 1.13 (1.01–1.30) | 0.041 |  | 1.15 (1.00–1.32) | 0.043 |  | 1.15 (1.01–1.32) | 0.040 |
| TyG-ABSI | 1.19 (1.09–1.31) | <.001 |  | 1.07 (0.95–1.21) | 0.290 |  | 1.10 (0.97–1.25) | 0.120 |  | 1.11 (0.98–1.27) | 0.100 |
| Cox |  |  |  |  |  |  |  |  |  |  |  |
| ABSI | 2.01 (1.78 - 2.28) | <.001 |  | 1.21 (1.06 - 1.38) | 0.005 |  | 1.20 (1.04 - 1.37) | 0.009 |  | 1.20 (1.05 - 1.38) | 0.010 |
| TyG-ABSI | 1.41 (1.23 - 1.62) | <.001 |  | 1.04 (0.89 - 1.22) | 0.606 |  | 1.02 (0.87 - 1.20) | 0.787 |  | 1.04 (0.88 - 1.24) | 0.650 |

**Note:** Model 1 (crude adjustment); Model 2 (adjusted for age and sex); Model 3 (further adjusted for ethnicity, education level, marital status, PIR, drinking status, smoking status, and physical activity level); and Model 4 (additionally adjusted for hypertension, and hyperlipidemia). CVD, cardiovascular disease; ABSI, a body shape index; TyG-ABSI, triglyceride glucose- a body shape index; HR, hazard ratios; CI, confidence interval; PIR, poverty-to-income ratio.

**Table S5.** **Calibration metrics (calibration slope and Brier score) for the Clinical, Clinical + ABSI, and Clinical + TyG-ABSI models in predicting 3-year and 5-year all-cause and cardiovascular disease mortality.**

| **Prediction Model** | **Brier Score** | **Calibration Slope** |
| --- | --- | --- |
| **All cuase mortality** |  |  |
| 3-Year |  |  |
| Clinical model | 0.024163 | 0.997 |
| Clinical model + ABSI | 0.024167 | 0.996 |
| Clinical model + TyG-ABSI | 0.024224 | 1.015 |
| 5-Year |  |  |
| Clinical model | 0.038975 | 0.988 |
| Clinical model + ABSI | 0.038767 | 0.995 |
| Clinical model + TyG-ABSI | 0.038843 | 0.981 |
| **CVD mortality** |  |  |
| 3-Year |  |  |
| Clinical model | 0.007389 | 0.945 |
| Clinical model + ABSI | 0.007393 | 0.955 |
| Clinical model + TyG-ABSI | 0.007404 | 0.924 |
| 5-Year |  |  |
| Clinical model | 0.013456 | 0.963 |
| Clinical model + ABSI | 0.013430 | 0.979 |
| Clinical model + TyG-ABSI | 0.013458 | 0.958 |

**Note:** The table presents the calibration slope and Brier score for predicting all-cause and CVD mortality at 3- and 5-year intervals. Three models are compared: 1) the Clinical model, adjusted for sex, age, race, education level, marital status, PIR, alcohol consumption, smoking status, physical activity, hypertension, and hyperlipidemia; 2) the Clinical +ABSI model; and 3) the Clinical + TyG-ABSI model. The calibration slope assesses the agreement between predicted and observed risks (ideal value = 1). The Brier score quantifies the overall prediction error, with lower values indicating better accuracy. ABSI, A Body Shape Index; TyG-ABSI, Triglyceride Glucose-ABSI.

Supplementary calculator

we have developed and deployed an interactive online risk calculator, accessible at: [**https://zhuzhengyang.shinyapps.io/risk-calculator/**](https://zhuzhengyang.shinyapps.io/risk-calculator/).

This calculator allows users, including clinicians and researchers, to directly translate our core findings into personalized risk assessments. The tool is designed with a simple interface where one inputs the patient's sex and their calculated ABSI or TyG-ABSI value. The calculator then instantly provides the corresponding sex-specific z-score. Crucially, for ABSI—which demonstrated significant threshold effects for mortality—the tool goes a step further. It automatically interprets the calculated z-score against the optimal cut-off values identified in our study (z > 0.624 for high all-cause mortality risk and z > 0.416 for high CVD mortality risk) and provides a clear, immediate risk stratification (e.g., "High Risk" or "Low Risk") for both outcomes. This functionality directly transforms the statistical thresholds we identified into an instant, actionable clinical interpretation.

STROBE checklis

|  | **Item No** | **Recommendation** | **Reported on Page No** |
| --- | --- | --- | --- |
| **Title and abstract** | 1 | (*a*) Indicate the study’s design with a commonly used term in the title or the abstract | 16 |
|  |  | (*b*) Provide in the abstract an informative and balanced summary of what was done and what was found | 22 |
| **Introduction** | | |  |
| Background/rationale | 2 | Explain the scientific background and rationale for the investigation being reported | 43 |
| Objectives | 3 | State specific objectives, including any prespecified hypotheses | 77 |
| **Methods** | | |  |
| Study design | 4 | Present key elements of study design early in the paper | 94 |
| Setting | 5 | Describe the setting, locations, and relevant dates, including periods of recruitment, exposure, follow-up, and data collection | 100 |
| Participants | 6 | (*a*) Give the eligibility criteria, and the sources and methods of selection of participants. Describe methods of follow-up | 106 |
|  |  | (*b*) For matched studies, give matching criteria and number of exposed and unexposed |  |
| Variables | 7 | Clearly define all outcomes, exposures, predictors, potential confounders, and effect modifiers. Give diagnostic criteria, if applicable | 140  123  152 |
| Data sources/ measurement | 8* | For each variable of interest, give sources of data and details of methods of assessment (measurement). Describe comparability of assessment methods if there is more than one group | 124  142 |
| Bias | 9 | Describe any efforts to address potential sources of bias | 187  196 |
| Study size | 10 | Explain how the study size was arrived at | 106 |
| Quantitative variables | 11 | Explain how quantitative variables were handled in the analyses. If applicable, describe which groupings were chosen and why | 191 |
| Statistical methods | 12 | (*a*) Describe all statistical methods, including those used to control for confounding | 208 |
|  |  | (*b*) Describe any methods used to examine subgroups and interactions | 220 |
|  |  | (*c*) Explain how missing data were addressed | 106 |
|  |  | (*d*) If applicable, explain how loss to follow-up was addressed | 179 |
|  |  | (*e*) Describe any sensitivity analyses | 220 |
| **Results** | | |  |
| Participants | 13* | (a) Report numbers of individuals at each stage of study—eg numbers potentially eligible, examined for eligibility, confirmed eligible, included in the study, completing follow-up, and analysed | 114 |
|  |  | (b) Give reasons for non-participation at each stage | 114 |
|  |  | (c) Consider use of a flow diagram | 114 |
| Descriptive data | 14* | (a) Give characteristics of study participants (eg demographic, clinical, social) and information on exposures and potential confounders | 233 |
|  |  | (b) Indicate number of participants with missing data for each variable of interest | 106 |
|  |  | (c) Summarise follow-up time (eg, average and total amount) | 234 |
| Outcome data | 15* | Report numbers of outcome events or summary measures over time | 335 |
| Main results | 16 | (*a*) Give unadjusted estimates and, if applicable, confounder-adjusted estimates and their precision (eg, 95% confidence interval). Make clear which confounders were adjusted for and why they were included | 265 |
|  |  | (*b*) Report category boundaries when continuous variables were categorized | 257 |
|  |  | (*c*) If relevant, consider translating estimates of relative risk into absolute risk for a meaningful time period |  |
| Other analyses | 17 | Report other analyses done—eg analyses of subgroups and interactions, and sensitivity analyses | 353 |
| **Discussion** | | |  |
| Key results | 18 | Summarise key results with reference to study objectives | 378 |
| Limitations | 19 | Discuss limitations of the study, taking into account sources of potential bias or imprecision. Discuss both direction and magnitude of any potential bias | 611 |
| Interpretation | 20 | Give a cautious overall interpretation of results considering objectives, limitations, multiplicity of analyses, results from similar studies, and other relevant evidence | 637 |
| Generalisability | 21 | Discuss the generalisability (external validity) of the study results | 451 |
| **Other information** | | |  |
| Funding | 22 | Give the source of funding and the role of the funders for the present study and, if applicable, for the original study on which the present article is based | 657 |

*Give information separately for exposed and unexposed groups.

**Note:** An Explanation and Elaboration article discusses each checklist item and gives methodological background and published examples of transparent reporting. The STROBE checklist is best used in conjunction with this article (freely available on the Web sites of PLoS Medicine at http://www.plosmedicine.org/, Annals of Internal Medicine at http://www.annals.org/, and Epidemiology at http://www.epidem.com/). Information on the STROBE Initiative is available at http://www.strobe-statement.org.

Supplementary Figure

**Figure S1.** **DCA Demonstrating the Superior Net Benefit of a Clinical Model Augmented with TyG-ABSI for Identifying Prevalent CKD.**


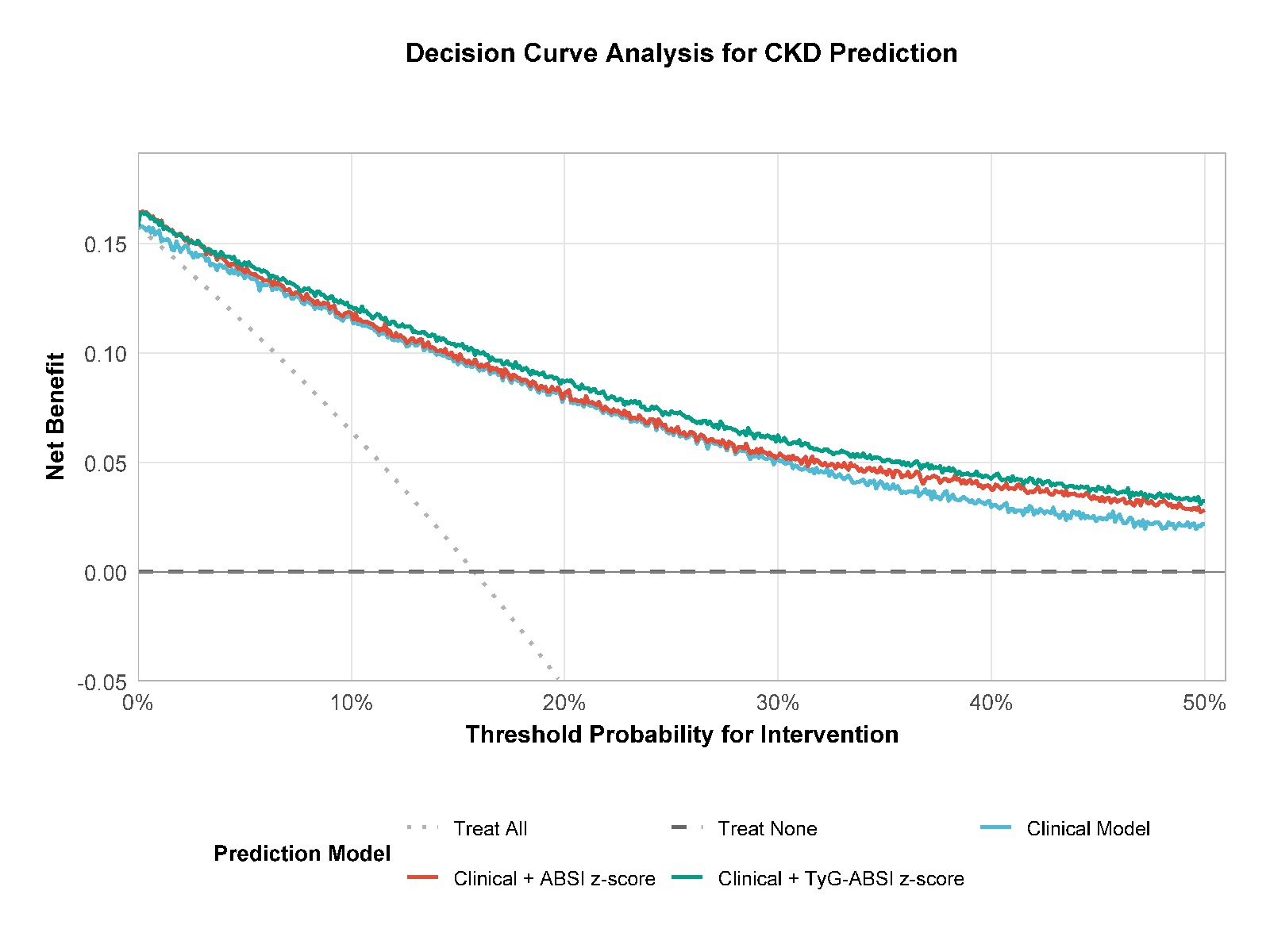


**Note**: DCA was performed to quantify the clinical net benefit of prediction models for prevalent CKD. Five distinct clinical strategies were evaluated and compared: 1) "Treat All", where all individuals are considered positive for CKD; 2) "Treat None", where no individuals are considered positive; 3) a "Clinical Model" based on a logistic regression model including sex, age, race, education level, marital status, PIR, alcohol consumption, smoking status, physical activity level, hypertension, and hyperlipidemia; 4) a "Clinical + ABSI z-score" model that augmented the Clinical Model with the standardized A Body Shape Index; and 5) a "Clinical + TyG-ABSI z-score" model that augmented the Clinical Model with the standardized Triglyceride Glucose-ABSI. The net benefit for each strategy was calculated across a continuous range of threshold probabilities (from 0% to 100%), which represent the minimum probability.

**Figure S2.** **Calibration curves for predicting all-cause mortality and CVD mortality at 3 and 5 years.**

**
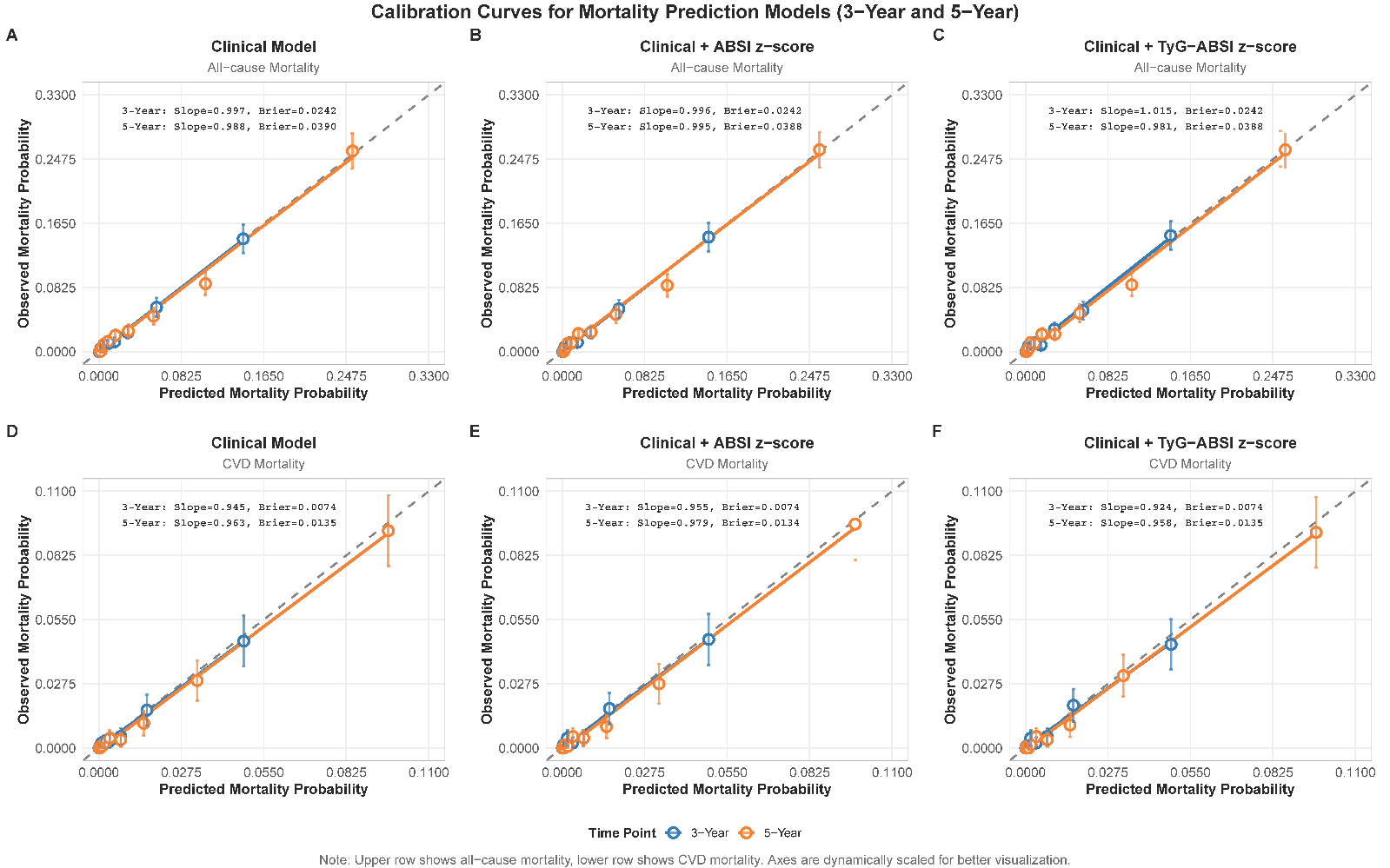
**

**Note:** Calibration plots for the Clinical model, Clinical + ABSI model, and Clinical + TyG-ABSI model. Panels A, B, and C display calibration for all-cause mortality at 3 and 5 years. Panels D, E, and F display calibration for CVD mortality at 3 and 5 years. The Clinical model includes sex, age, race, education level, marital status, PIR, alcohol consumption, smoking status, physical activity, hypertension, and hyperlipidemia. The dashed line represents the ideal calibration (45-degree line). ABSI, A Body Shape Index; TyG-ABSI, Triglyceride Glucose-ABSI.

**Figure S3.** **Comparison of calibration slopes and Brier scores for models predicting all-cause and CVD mortality.**

**
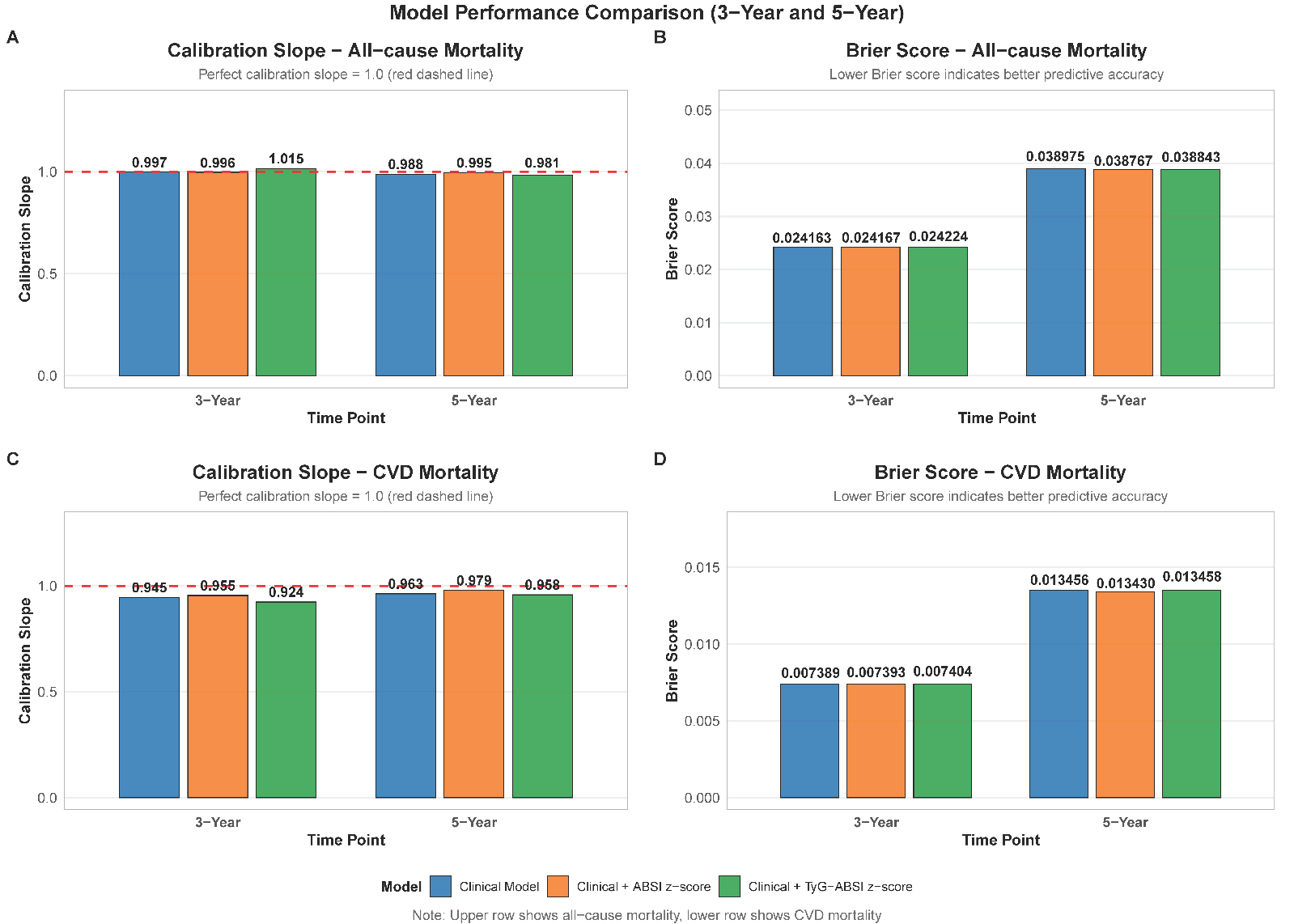
**

**Note:** Comparative performance of the Clinical model, Clinical + ABSI model, and Clinical + TyG-ABSI model. Panel A shows calibration slopes for all-cause mortality. Panel B shows Brier scores for all-cause mortality. Panel C shows calibration slopes for CVD mortality. Panel D shows Brier scores for CVD mortality. The calibration slope measures how well the model's predictions match the observed outcomes (ideal value=1). The Brier score measures overall prediction error (lower scores indicate better accuracy). The Clinical model includes sex, age, race, education level, marital status, PIR, alcohol consumption, smoking status, physical activity, hypertension, and hyperlipidemia. ABSI, A Body Shape Index; TyG-ABSI, Triglyceride Glucose-ABSI.
